# Phytochemistry, Pharmacology, and Nutraceutical Profile of *Carissa* Species: An Updated Review

**DOI:** 10.3390/molecules26227010

**Published:** 2021-11-20

**Authors:** Jyoti Dhatwalia, Amita Kumari, Rachna Verma, Navneet Upadhyay, Ishita Guleria, Sohan Lal, Shabnam Thakur, Kasahun Gudeta, Vikas Kumar, Jane C.-J. Chao, Somesh Sharma, Ashwani Kumar, Amanda-Lee Ezra Manicum, José M. Lorenzo, Ryszard Amarowicz

**Affiliations:** 1 School of Biological and Environmental Sciences, Faculty of Sciences, Shoolini University, Solan 173212, HP, India; dhatwaliajyoti3096@gmail.com (J.D.); rachnac83@gmail.com (R.V.); ishita.thakur93@gmail.com (I.G.); Sohanlal48510@gmail.com (S.L.); Shabnamthakur780@gmail.com (S.T.); kasahungudeta40@gmail.com (K.G.); 2 School of Pharmaceutical Sciences, Shoolini University, Solan 173212, HP, India; navneetqa@gmail.com; 3 Department of Applied Biology, Adama Science and Technology University, Adama, P.O. Box 1888, Ethiopia; 4 University Institute of Biotechnology, Chandigarh University, Gharuan, Mohali, Punjab 140413, India; vikaskmr59@gmail.com; 5 School of Nutrition and Health Sciences, College of Nutrition, Taipei Medical University, 250 Wu-Hsing Street, Taipei 11031, Taiwan; chenjui@tmu.edu.tw; 6 Nutrition Research Center, Taipei Medical University Hospital, 252 Wu-Hsing Street, Taipei 11031, Taiwan; 7 School of Bioengineering and Food Technology, Shoolini University of Biotechnology and Management, Solan 173229, HP, India; sharmawine@gmail.com; 8 Patanjali Herbal Research Department, Patanjali Research Institute, Haridwar, Uttarakhand 249405, India; ashu5157@gmail.com; 9 Department of Chemistry, Tshwane University of Technology, Pretoria 0183, South Africa; ManicumAE@tut.ac.za; 10 Centro Tecnológico de la Carne de Galicia, Parque Tecnológico de Galicia, 32900 San Cibrao das Viñas, Spain; jmlorenzo@ceteca.net; 11 Área de Tecnología de los Alimentos, Facultad de Ciencias de Ourense, Universidad de Vigo, 32004 Ourense, Spain; 12 Institute of Animal Reproduction and Food Research, Polish Academy of Sciences, 10-748 Olsztyn, Poland

**Keywords:** *Carissa*, phytochemistry, bioactive compounds, nutraceutical profile, pharmacological activity

## Abstract

*Carissa*, a genus of the Apocynaceae family, consists of evergreen species, such as shrubs as well as small trees that are native to Asia, Africa, and Oceania’s subtropical and tropical regions. Most of the *Carissa* species are traditionally used to treat various diseases, such as chest pain, headaches, gonorrhoea, rheumatism, syphilis, oedema, rabies, stomach pain, hepatitis, cardiac diseases, and asthma. The pharmacological studies on *Carissa* species revealed its antioxidant, antimicrobial, anticancer, cardioprotective, antipyretic, analgesic, wound healing, anticonvulsant, antiarthritic, adaptogenic, anti-inflammatory, and antidiabetic activities, thus validating its use in indigenous medicine systems. The review article summarised the comprehensive literature available, including morphology, indigenous uses, bioactive composition, nutraceutical, and pharmacological activities of *Carissa* species. A total of 155 research papers were cited in this review article. The *Carissa* fruits are rich in dietary fibre, lipids, proteins, carbohydrates, vitamin C, and macro- and micro-elements. A total of 121 compounds (35 polyphenols (flavonoids and phenolic acids), 30 lignans, 41 terpenoids, 7 steroids, 2 coumarins, and 6 cardiac glycosides) have been extracted from *C. spinarum*, *C. carandas,* and *C. macrocarpa*. Among all chemical constituents, lupeol, carissol, naringin, carisssone, scopoletin, carissaeduloside A, D, J, carandinol, sarhamnoloside, carissanol, olivil, carinol, 3β-hydroxyolean-11-en-28,13β-oilde, ursolic acid, and carissone are the key bioactive constituents responsible for pharmacological activities of genus *Carissa*. The gathered ethnopharmacological information in the review will help to understand the therapeutic relevance of *Carissa* as well as paving a way for further exploration in the discovery of novel plant-based drugs.

## 1. Introduction

*Carissa* is one of the most important genera in the *Apocynaceae* family of order Gentianales. It consists of evergreen shrubs or small trees native to the subtropical and tropical regions of Asia, Africa, and Oceania [1]. The genus consists of approximately 85 species, but out of these only 8 have accepted names, whereas other species are either synonyms of these 8 species or assigned to other genera [2]. The species with their accepted names are *C. bispinosa* (L.) Desf. ex Brenan, *C. boiviniana* (Baill.) Leeuwenb., *C. carandas* L., *C. haematocarpa* (Eckl.) A.DC., *C. macrocarpa* (Eckl.) A.DC., *C. pichoniana* Leeuwenb., *C. spinarum* L., and *C. tetramera* (Sacleux) Stapf [2]. Traditionally, *Carissa* plants have been used for the treatment of a variety of diseases, such as headache, syphilis, chest discomfort, gonorrhoea, malaria, arthritis, and rabies, since time immemorial [3,4,5,6,7,8,9]. Besides these, they are also used against sickle cell anaemia, ulcers, and worm infections [1]. The fruits of the genus are rich in dietary fibre, lipids, protein, carbohydrates, and macro- and micro-elements, and as a result, they play a crucial role in promoting human health [10].

Previous comprehensive reports on *Carissa* species are either available only on the traditional uses and phytochemistry [11] or on the research carried out on single species [12,13]. This review provides complete information on various aspects of four *Carissa* species, e.g., *C*. *carandas*, *C. macrocarpa* [syn. *C. grandiflora* (E. Mey.) A. Dc], *C. bispinosa,* and *C. spinarum* (syn. *C. opaca* Stapt ex Haines, *C. edulis* Vahl, *C. lanceolata* R. Br, and *C. congesta* Wt.), emphasizing taxonomy, ethnomedicinal and nutraceutical uses, phytochemistry, and pharmacological activities, covering the periods from 1951 to 2021. This information may provide opportunities for researchers around the world to investigate the unexplored species of the genus by isolating new bioactive phytoconstituents.

### 1.1. Research Methdology

The scientific literature was searched through various databases, such as Scopus, Google Scholar, Science Direct, The Plant List, Plant of the World Online, and PubMed. The current review article contains the research carried out on *Carissa* species in the fields of nutrition, phytochemistry, and pharmacology over the period from 1950 to 2021. The chemical structure of compounds was drawn using Chem draw software. The scientific names of plant species were validated from The Plant List database [2], and their distribution was taken from the Plant of the World Online [14].

### 1.2. Distribution of Carissa Species

**Figure 1:**
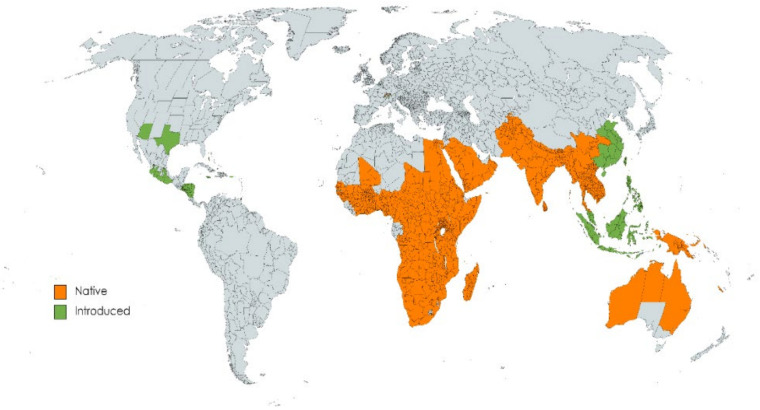
Geographical distribution of *Carissa* species (Created with mapchart.net).

*Carissa’s* native range is Africa to Indo-China, Australia to New Caledonia, and has been introduced into the Bahamas, China, Central America, Jamaica, Indonesia, Malaya, Mexico, Nicaragua, the Philippines, Taiwan, Trinidad-Tobago, and the USA [14] (Figure 1).

## 2. General Morphology

**Figure 2:**
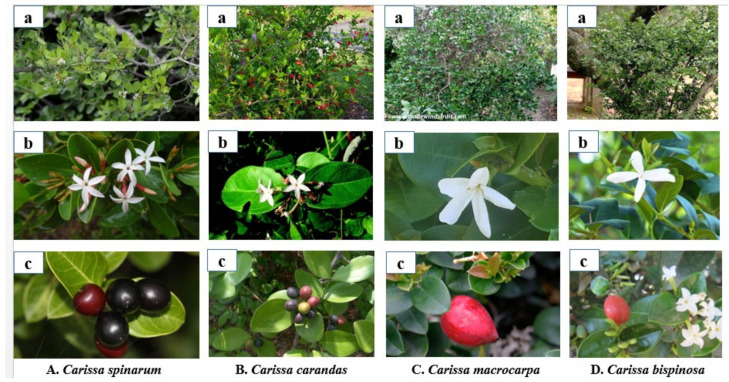
Representative pictures showing morphology of *Carissa spinarum* (**A**), *C. carandas* (**B**), *C. macrocarpa* (**C**), and *C. bispinosa* (**D**): a—whole plant; b—flower; c—fruit. Sources: *C. spinarum* (http://www.plantsoftheworldonline.org, accessed on 10 November 2021); *C. carandas* plant (http://chengailimfruittrees.blogspot.com, accessed on 10 November 2021); *C. carandas* flowers (https://indiabiodiversity.org, accessed on 10 November 2021); *C. carandas* fruits (http://tropical.theferns.info, accessed on 10 November 2021); *C. macrocarpa* plant and fruit (http://www.tradewindsfruit.com, accessed on 10 November 2021); *C. macrocarpa* flower (http://www.plantsoftheworldonline.org, accessed on 10 November 2021); *C. bispinosa* plant (http://natureswow2.blogspot.com, accessed on 10 November 2021); *C. bispinosa* flowers and fruits (https://treesa.org, accessed on 10 November 2021).

The plants are evergreen, multi-stemmed (*C. macrocarpa*), sprawling (*C. carandas*), and dense bush or rambling (*C. bispinosa*) shrubs (*C. spinarum*), which grow as a small tree up to 1–5 m in height. The stem is branched, glabrous, greenish, spiny, and rich in white latex. Leaves are in opposite-decussate arrangement, green, simple, obovate or oblanceolate (*C. carandas*), ovate or rounded (*C. spinarum, C. bispinosa*), thick coriaceous (*C. macrocarpa*), glabrous with obtuse or emarginated (*C. carandas*), acute or rounded (*C. spinarum*), apex and acute to cuneate (*C. carandas*), cuneate or rounded (*C. spinarum*) base. Flowers begin to bloom between February and April in *C. spinarum*, March–April in *C. carandas*, May in *C. macrocarpa,* and October to March in *C. bispinosa*. Flowers are small, fragrant, white inside, and pink to red on the outside (*C. spinarum*), white (*C. carandas*, *C. macrocarpa*), jasmine-scented (*C. macrocarpa*), white or tinged pink (*C. bispinosa*), glossy, tubular, and 5-lobed. Fruiting takes place throughout the year. The fruits are globose or ellipsoid (*C. carandas, C. spinarum*), ovoid (*C. bispinosa*), or pointed (*C. macrocarpa*) in shape, red (*C. macrocarpa, C. bispinosa*), dark blue (*C. carandas*), or red to black (*C. spinarum*) when fully ripened. Seeds are llipsoid (*C. carandas*), lanceolate (*C. spinarum*), and black or brown in colour [8,15,16,17,18,19,20] (Figure 2).

## 3. Traditional Uses

*Carissa* genus is reported with numerous ethnomedicinal uses (Table 1). The plant parts such as leaf, bark, stem, fruit, and roots have found different uses in the management and treatment of diseases such as fever, cold and cough, ulcer, toothache, rheumatism, diarrhoea, epilepsy, anaemia, and cardiovascular diseases [8,15,16,17,18,19,20]. The whole plant of *C. spinarum* is traditionally used in India, Ethiopia, and other African countries for the remedy of venereal respiratory and gastrointestinal infections [21,22,23,24,25,26,27,28,29,30,31], fever, jaundice, hepatitis, cardiac diseases, asthma [22], gonorrhoea, stomach-ache, chickenpox, wound healing, rabies, and also as an antidote to snake bites [32,33]. The leaves of *C. spinarum* are also used as a mosquito repellent [32]. According to Chandra et al. [22], the consumption of *C. spinarum* fruits promotes good health and well-being and reduces the risk of chronic disease, whereas roots can provide new leads for antimicrobial drugs [34,35].

The role of the *C. carandas* whole plant has been found to be effective in the treatment of liver diseases [48], convulsions [21], asthma, tuberculosis, scabies, and wound healing [41,42,49]. Fruits of *C. macrocarpa* have shown their essential role in the remedy of human immunodeficiency virus (HIV) and hepatitis [8,45].

## 4. Nutraceutical Profile of *Carissa* Species

The *Carissa* fruits are rich in fibres, lipids, proteins, carbohydrates, and macro- and micro-nutrients, which are essential to build and maintain strong bones and to retain normal functioning of the heart, kidney, muscles, and nerves [10,46,50]. The fruits are rich in nutritive compounds that improve taste and also prolong the shelf-life of food products. Ripe fruits are eaten raw and used for making the excellent quality cakes, ice cream, jams, squash, and jelly, which resemble gooseberry in flavour, whereas unripe fruits are used for making chutney, pickles, and candies. *C. carandas* fruits (ripe) have also been documented to be used as a natural food decolourant cum nutraceutical supplement in the lime sharbat, named “Lalima” [51]. The nutraceutical values of *Carissa* plants are summarised in Table 2.

## 5. Phytochemistry

**Figure 3:**
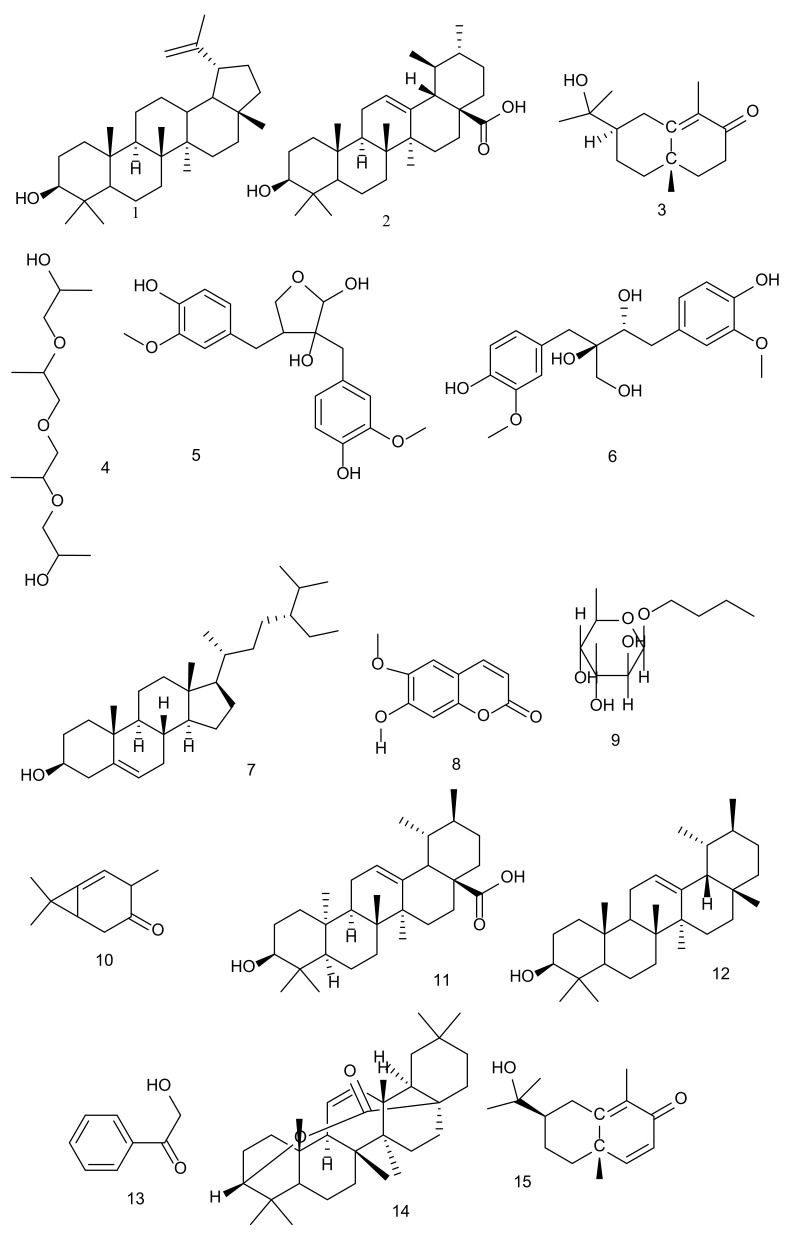

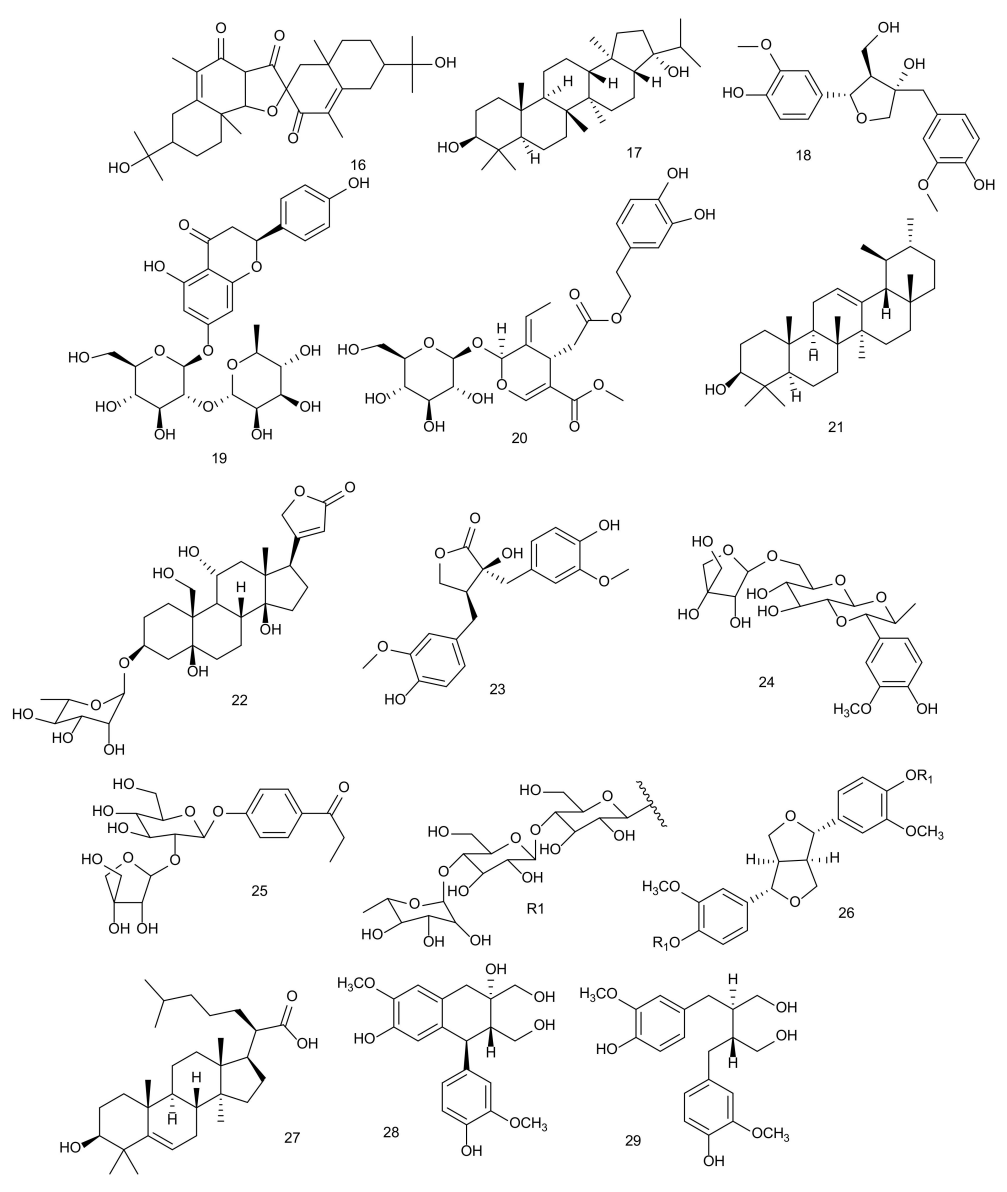

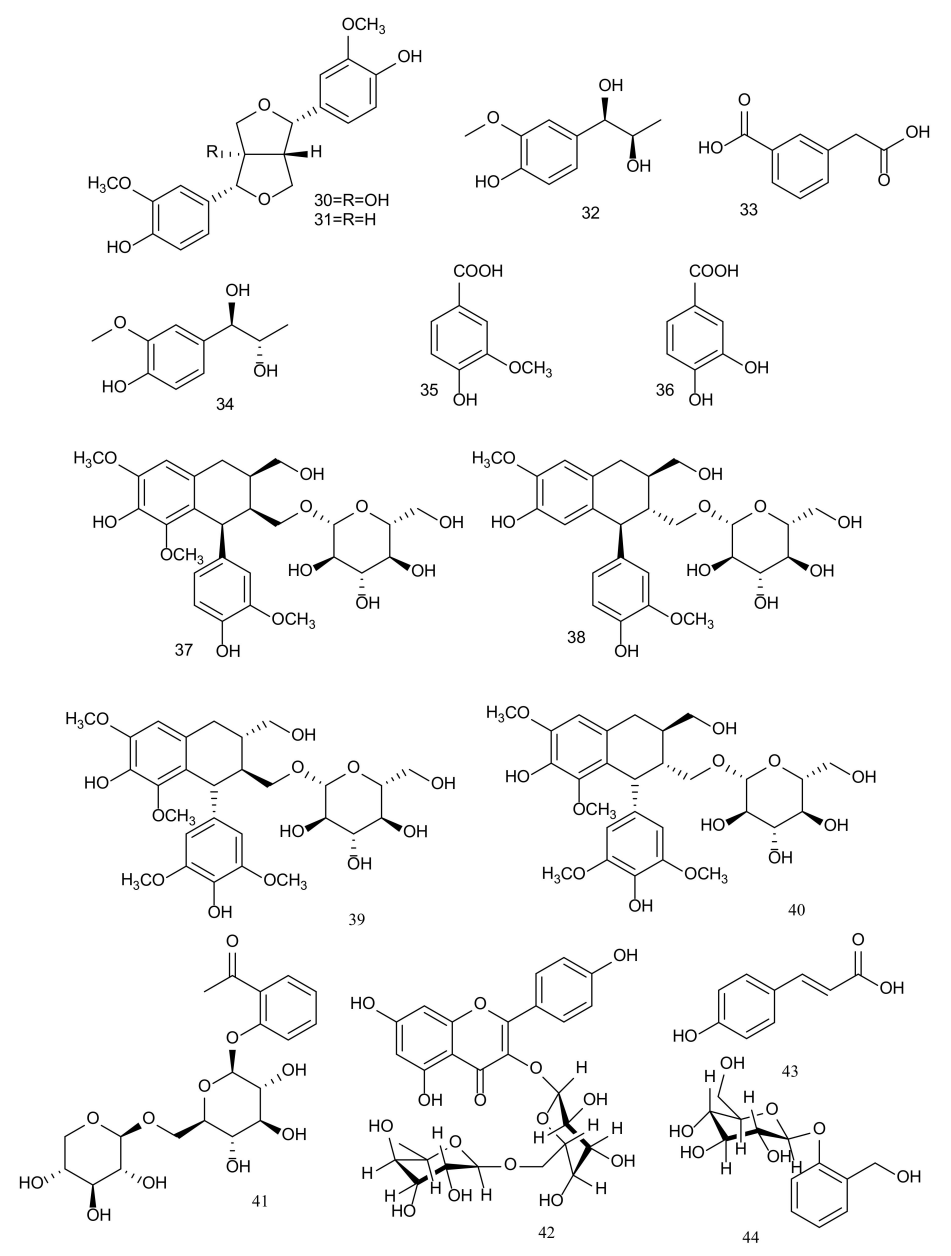

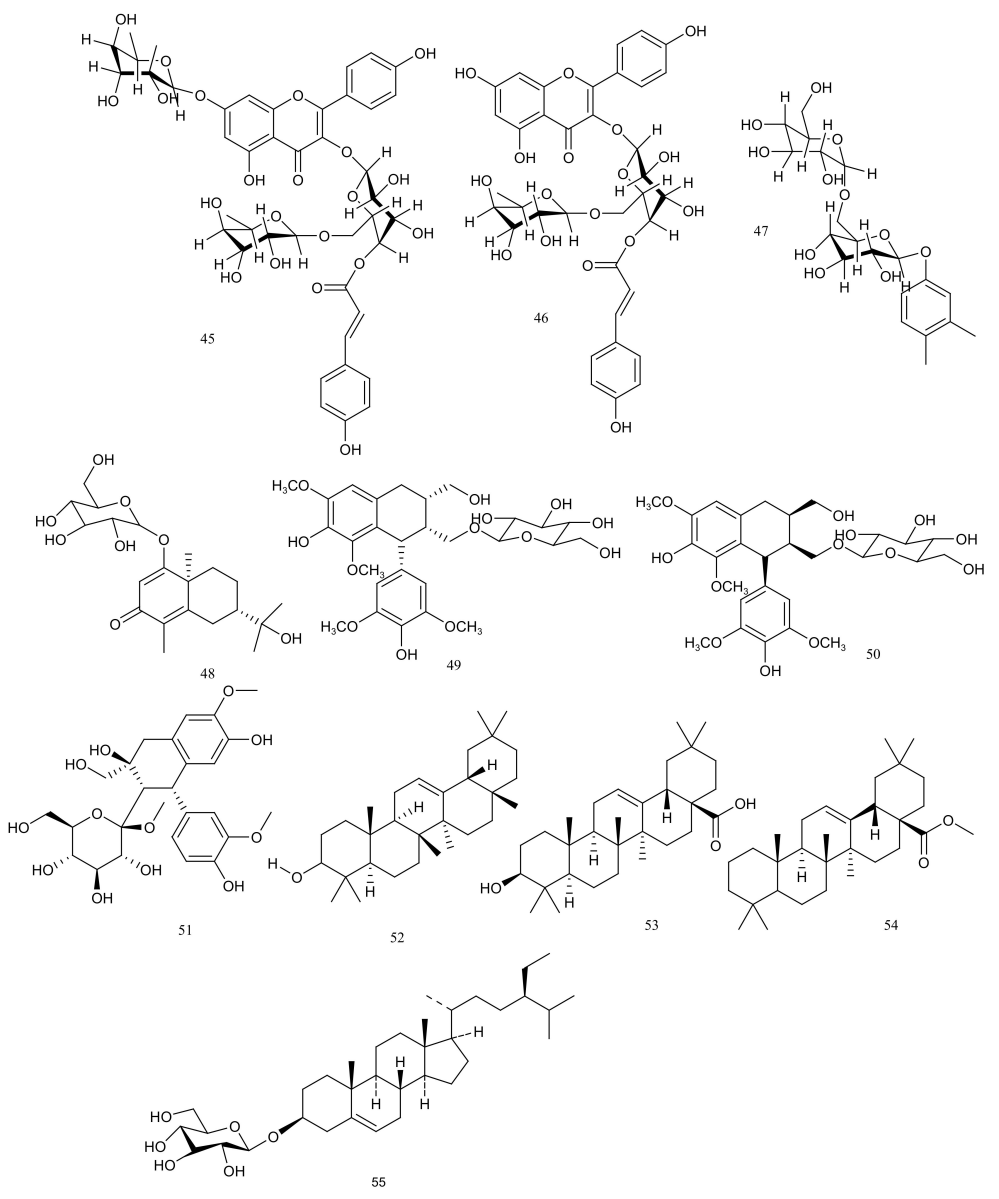
Chemical structure of biologically active isolated compounds of *Carissa* species: lupeol (**1**), ursolic acid (**2**), carissone (**3**), caredulis,-{1-[2-(2 hydroxypropoxy) propoxy] propan-2-yloxy} (**4**), carissanol (**5**), carinol (**6**), β-sitosterol (**7**), scopoletin (**8**), butyl-*O*-α-l-rhamnoside (**9**), carenone (**10**), carissic acid (**11**), carissol (**12**), 2-hydroxyacetophenone (**13**), 3β-hydroxyolean-11-en-28,13β-olide (**14**), dehydrocarissone (**15**), carindone (**16**), carandinol (**17**), olivil (**18**), naringin (**19**), oleuropein (**20**), ɑ-amyrin (**21**), sarhamnoloside (**22**), nortrachelogenin (**23**), carissaedulosides A (**24**), carissaedulosides d (**25**), carissaedulosides J (**26**), lanost-5-en-3b-ol-21-oic acid (**27**), (+)-cycloolivil (**28**), (−)-secoisolariciresinol (**29**), R=OH ((+)-8-hydroxypinoresinol (**30**), R=H ((+)-pinoresinol (**31**), erythro-1-(3-methoxy-4-hydroxy-phenyl)-propan-1,2-diol (**32**), 3-carboxy methyl-benzoic acid (**33**), threo-1-(3methoxy-4-hydroxy-phenyl)-propan-1,2-diol (**34**), vanillic acid (**35**), protocatechuic acid (**36**), (6*R*,7*S*,8*S*)-7a-[(β-d-glucopyranosyl)-oxy]1-methoxy isolariciresinol (**37**), (+)-isolariciresinol3a-*O*-β-d-glucopyranoside (**38**), (−)lyoniresinol3α-*O*-β-d-glucopyranoside (**39**), (+)-lyoniresinol3α-*O*-β-d-glucopyranoside (**40**), acetophenone-2-*O*-βxylopyranosyl-(1→6)-*O*-β-glucopyranoside (**41**), kaempferol-3-*O*-robinobioside (**42**), *p*-coumaric acid (**43**), salicin (**44**), kaempferol-3-*O*-α-l-rhamopyranosyl (**45**), variabiloside E (**46**), 3,4-dimethylphenol β-gentiobioside (**47**), carandoside (**48**), (6*S*,7*R*,8*R*)-7a-[(β-glucopyranosyl) oxy]lyoniresinol (**49**), (6*R*,7*S*,8*S*)-7a-[(β-glucopyranosyl)oxy]lyoniresinol (**50**), [(1*S*,2*S*,3*S*)-1,2,3,4-tetrahydro-3,7-dihydroxy-1-(4-hydroxy-3-methoxyphenyl)-3-(hydroxymethyl)-6methoxy-2-naphthalen-yl] methyl β-d-gluco-pyranoside (**51**), β-amyrin (**52**), methyl oleanate (**53**), oleanolic acid (**54**), β-sitosterol-3-*O*-β-d-glucopyranoside (**55**).

Genus *Carissa* is rich in different classes of primary and secondary metabolites, including carbohydrates, lipids, proteins, and phenolics including flavonoids and tannins, terpenoids, coumarins, lignins, glycosides, tannins, and steroids [22,25,44,55,56,57,58,59,60,61,62,63,64,65]. Extensive work has been carried out by researchers to distinguish several compounds from the leaves, stems, roots, and wood of the *Carissa* species. A total of 93 compounds from *C. spinarum* (27 polyphenols, 27 lignans, 23 terpenoids, 8 steroids, 2 coumarins, and 6 cardiac glycosides)*,* 28 from *C. carandas* (2 polyphenols, 4 lignans, 20 terpenoids, and 2 steroids), and 11 from *C. macrocarpa* (6 polyphenols and 5 terpenoids) have been extracted (Figure 3).

### 5.1. Polyphenols (Phenolic Acids and Flavonoids)

From the genus *Carissa,* a total of 35 polyphenols including phenolic acids and flavonoids were isolated. The caffeic acid and carissone were isolated from the roots of *C. spinarum* [66]. Five phenolic compounds (two phenolics (caffeic acid methyl ester and chlorogenic acid-1-ethyl ether-1-methyl ester), two flavonols (kaempferol and rutin), and one flavanol glycoside (quercetin-3-*O*-glucoside-7,3′,4′-trimethyl ether)) were isolated from the aerial parts of *C. edulis* (syn. *C. spinarum*) by Al-Youssaf and Hassan [67]. Likewise, four flavonol glucosides, namely kaempferol 3-*O*-β-d glucopyranoside, quercetin-3-*O*-β-d glucopyranoside, rhamnetin-3-*O*-β-d-glucopyranoside, and isorhamnetin-3-*O-*β-d-glucopyranoside, and one derivative of phenolic acid (3-*O*-acetyl chlorogenic acid) were eluted for the first time from the ethyl acetate fraction of aerial parts of *C. edulis* (syn. *C. spinarum*) by Al-Youssaf and Hassan [68]. Similarly, from the butanol fraction of aerial parts of *C. edulis* (syn. *C. spinarum*), Al-Youssaf and Hassan [69] isolated one flavonol glucoside (isoorhamnetin-3-*O*-β-d-glucopyranoside-rhamnopyranoside) and two other phenolic compounds (caredulis,1-{1-[2-(2 hydroxypropoxy) propoxy]} propan-2-yloxy (**4**) and butyl-*O*-α-l-rhamnoside (**9**)). A flavanone, naringin (**19**), was isolated from the methanolic leaves’ extract of *C. carandas* by El-Desoky et al. [70], and the authors have also reported their anti-inflammatory and antioxidant activities (Table 3). The volatile phenolic compound (2-hydroxyacetophenone (**13**)), having antibacterial potential (Table 4), was reported for the first time from *C. lanceolate* (syn. *C. spinarum*) by Hettiarachchi et al. [39] (Table 4). Three flavonoids (kaempferol-3-*O*-robinobioside (**42**), kaempferol-3-*O*-α-l-rhamnopyranosyl (1–6)(4″-*p*-coumaroyl) β-d-galactopyranoside 7-*O*-α-l-rhamnopyrano-side (**45**), and variabiloside E (**46**)), one phenolic acid (*p*-coumaric acid (**43**)) along with two phenolic glycosides (salicin (**44**) and 3,4-dimethylphenol β-gentiobioside (**47**)) were isolated from leaves of *C. macrocarpa,* and all compounds have been screened for cytotoxicity activity [71] (Table 5). Begum et al. [49] extracted 4-hydroxybenzoic acid (a phenolic acid) from the leaves of *C. carandas*. Two flavones (epigenin and luteolin) have been extracted by Parveen et al. [72] from the aerial parts of *C. opaca* (syn. *C. spinarum*). In the year 2021, Liu et al. [73] isolated a total of ten phenolic compounds, such as (6*R*,7*S*,8*S*)-7a-[(β-d-glucopyranosyl)-oxy]-1-methoxyisolariciresinol (**37**), (+)-isolariciresinol3a-*O*-β-d-glucopyranoside (**38**), (+)-lyoniresinol3α-*O*-β-d-glucopyranoside (**40**), (−)-lyoniresinol3α-*O*-β-d-glucopyranoside (**39**), acetophenone-2-*O*-βxylopyranosyl-(1→6)-*O*-β-glucopyranoside (**41**), erythro-1-(3- methoxy-4-hydroxy-phenyl)-propan-1,2-diol (**32**), threo-1-(3-methoxy-4-hydroxy-phenyl)-propan-1,2-diol (**34**), 3-carboxymethyl-benzoic acid (**33**), protocatechuic acid (**36**), and and vanillic acid (**35**), from the root bark of *C. spinarum.* The authors have also screened these compounds for their antioxidant activity [73] (Table 3).

### 5.2. Lignans

A total of thirty lignans have been reported from different species of *Carissa*. Rao et al. [78] isolated a total of seven lignans (coniferaldehyde, pinoresinol (**31**), nortrachelogenin (**23**), carissanol (**5**), secoisolariciresinol (**29**), carinol (**6**), and olivil (**18**)) from the chloroform extract of *C. spinarum* stem, and out of these compounds, only carissanol (**5**) and carinol (**6**) showed antioxidant potential. Similarly, Wangteeraprasert and Likhitwitayawuid [56] eluted three lignans ((6*R*,7*S*,8*S*)-7a-[(b-glucopyranosyl)oxy]lyoniresinol, (6*S*,7*R*,8*R*)-7a-[(β-glucopyranosyl)oxy] lyoni-resinol (**49**), carissanol (**5**), and nortrachelogenin (**23**)) from the stem of *C. carandas.* These isolated compounds were also screened by the authors for their antioxidant activity [56] (Table 3). From the *C. lanceolate* (syn. *C. spinarum*) root, carinol (**6**) was reported by Hettiarachchi et al. [39]. Additionally, a total of eleven lignans (carinol (**6**), olivil (**18**), cycloolivil (**28**), 8-hydroxypinoresinol (**30**), secoisolariciresinol (**29**), digitoxigenin-3-*O*-β-d-digitalopyranoside, nortrachelogenin (**23**), evomonoside, scopoletin (**8**), (+)-pinoresinol (**31**), and carissanol (**5**)) have been extracted from the methanol extract of *C. spinarum* stem, followed by the analysis of their free radical scavenging (DPPH assay) and cytotoxic activities [23] (Table 3 and Table 5). Other lignans, nortrachelogenin (**23**), carinol (**6**), olivil (**18**), and carissanol (**5**), were isolated from the methanolic root extract of *C. edulis* (syn. *C. spinarum*) by Achenbach et al. [99].

Kaunda et al. [94] isolated three furofuran lignans (carissaeduloside H, carissaeduloside I, and carissaeduloside J (**26**)) together with 12 analogues (sarhamnoloside (**22**), [(1*S*,2*S*,3*S*)-1,2,3,4-tetrahydro-3,7-dihydroxy-1-(4-hydroxy-3-methoxyphenyl)-3-(hydroxymethyl)-6-methoxy-2-naphthalen-yl] methyl β-d-glucopyranoside (**51**), (−)-lyoniresinol 9-*O*-β-d-glucopyranoside, (−)-lyoniresinol 9′-*O*-d-glucopyranoside, guaiacylglycerol, (+)-1-acetoxypinoresinol-4′-β-d-glucoside4″-*O*-methyl ether, (+)-1-acetoxypinoresinol4′-β-d-glucoside, 1-(1-hydroxyethyl)-2-(6-(1-hydroxyethyl) phenoxy)benzene, scopoletin (**8**), khaephuoside A, 3,4-dimethoxyphenyl 2-*O*-β-d-apiofuranosyl-(1→2)-β-d-glucopyranoside, and markhamioside F) from the root bark of *C. edulis* (syn. *C. spinarum*). Out of these compounds, sarhamnoloside (**22**) and [(1*S*,2*S*,3*S*)-1,2,3,4-tetrahydro-3,7-dihydroxy-1-(4-hydroxy-3-methoxyphenyl)-3-(hydroxyl-methyl)-6-methoxy-2-naphthalen-yl] methyl β-d-glucopyranoside (**51**) exhibited anticancer activity against lung and breast cancer cell lines [94] (Table 5).

### 5.3. Terpenoids

A total of 41 terpenes and terpenoids were reported from the *Carissa* species. In 1985, the compound carissol (**12**), an epimer of α-amyrin (**21**), was isolated from *C. carandas* [100]. The methanolic extract from the root of *C. edulis* (syn. *C. spinarum*) was characterised with seven sesquiterpenes (eudesmane-type and a novel germacrane derivate), e.g., carissone, cryptomeridiol, β-eudesmol, germacrenone, 6β-carissanol, 6α-carissanol, and α-carissanol [101]. Whereas *C. carandas* fruits were rich in two triterpenes (carissic acid (**11**) and carissic acid methyl ester) [102]. From the dichloromethane extract of the wood of *C. lanceolata* (syn. *C. spinarum*), three terpenoids (carissone (**3**), dehydrocarissone (**15**), and carindone (**16**)) have been isolated by Lindsay et al. [1] and also reported for their antibacterial activity (Table 4). Four pentacyclic triterpenoids (oleanolic acid (**54**), ursolic acid (**2**), carissin, and 3-β-hydroxy-27-*p*-E-coumaroyloxyurs-12-en-28-oic acid) were isolated for the first time by Siddiqui et al. [102] from the leaves of *C. carandas*. Similarly, Rao et al. [78] isolated germacrane sesquiterpenes (carenone (**10**), 3′-(4′′-methoxyphenyl)-3′-oxo-propionylhexadecanoate, and germacrenone) from the *C. spinarum* stem.

From the petroleum ether root extract of *C. carandas,* three triterpenoids (lupa-12,20(29)-dien-3b,28-diol, urs-12-ene-3β,22β-diol, and ursolic acid (**2**)) were isolated [103]. One new compound, carandoside (**48**), was extracted and analysed for its antioxidant activity from the stem of *C. carandas* [56] (Table 4). The compounds lupeol (**1**), 16 β-hydroxy betulinic acid, and α-amyrin (**21**) were reported for the first time from the roots of *C. carandas* by Hegde and Joshi [104,105]. Tolo et al. [84] isolated four terpenoids (lupeol (**1**), oleuropein (**20**), carissol (**12**), and β-amyrin) from the root bark of *C. edulis* (syn. *C. spinarum*) and screened these compounds for antiviral activity [106]. Likewise, Moodley et al. [8] isolated four pentacyclic oleanane triterpenes (β-amyrin (**52**), methyl oleanolate (**53**), oleanolic acid (**54**), and 3β hydroxyolean-11-en-28,13β-olide (**14**)) from the fruits of *C. macrocarpa*, while the ursane-type triterpene, ursolic acid (**2**), was isolated from the leaves of *C. macrocarpa* and also reported by the authors for antibacterial activity (Table 4).

Two triterpenoids, namely β-amyrin and lupeol (**1**), have been isolated from *C. edulis* (syn. *C. spinarum*) [68]. Carissone (**3**) (a terpenoid) was isolated by Joshi and Boyce [107], Hettiarachchi et al. [39], and Wangteeraprasert et al. [23] from the roots of *C. congesta* (syn. *C. spinarum*), the roots of *C. lanceolate,* and the stem of *C. spinarum*, respectively. The compound has also been screened for free radical scavenging activity (DPPH) and cytotoxic activity [23] (Table 3 and Table 5).

Similarly, Karunakar et al. [108] reported triterpenoids such as lupeol (**1**), ursolic acid (**2**), 17-hydroxy-11-oxo-nor-β-amyrone, and urs-12-ene-3β,22β-diol-17-carboxylic acid from a petroleum ether extract of *C. spinarum* roots. Whereas lanostane triterpenoid (lanost-5-en-3*β*-ol-21-oic acid (**27**)) was extracted from the ethanolic extract of the fruit of *C. carandas* by Arif et al. [109], and the authors also reported on its apoptogenic activity. Carandinol (**17**), a triterpene, was isolated for the first time by Begum et al. [41] from the leaves of *C. carandas*, along with three known compounds, betulinic acid, oleanolic acid, and ursolic acid (**2**). Among all these compounds, only carandinol (**17**) exhibited a cytotoxic effect against cancer cell lines [41] (Table 5). Lupeol (**1**), carissone (**3**), and oleanolic acid were isolated from the roots of *C. carandas* and screened for anti-inflammatory activity [110].

Parveen et al. [111] isolated two new nor-triterpenoids (30-nor-2a,3b-dihydoxyurs-12-ene and 30-nor-2a,3b,23-trihydroxyurs-12-ene) from aerial parts of *C. opaca* (syn. *C. spinarum*). In the year 2017, Parveen et al. [72] also isolated one more triterpene (ursolic acid (**2**)) from the aerial parts of *C. opaca*. Whereas, from the aerial parts of *C. opaca* (syn. *C. spinarum*), new nortriterpenoids noropacursane, 6-methoxy-7-hydroxycoumarin, lupeol (**1**), and 4-Ketopinoresinol have been extracted and characterised by Parveen et al. [112]. Additionally, Bano et al. [95] for the first time isolated ursane-type triterpene (sabiracin, para hydroxy benzaldehyde, ursolic acid (**2**), carissin, and 22a-hydroxyursolic acid) from fresh leaves of *C. carandas* and reported on their anticancer activity (Table 5).

### 5.4. Steroids

A total of seven steroids were isolated from the *Carissa* species. Hegde and Joshi [105] reported β-sitosterol (**7**) from chloroform extracts of *C. carandas* roots, while (3β,5α)-Stigma-7,16-dien-3-ol, sitosterol glucoside, and β-sitosterol (**7**) were isolated from the roots of *C. congesta* (syn. *C. spinarum*) [107]. The bioactive compounds named stigmasterol glucoside, β-sitosterol (**7**), and β-sitosterol glucoside were isolated by AI-Youssaf and Hassan [69] from the aerial parts of *C. edulis* (syn. *C. spinarum*). From the methanol extract of the aerial parts of *C. opaca,* β-sitosterol (7) and β-sitosterol 3-*O*-β-d-glucopyranoside (**55**) have been extracted [72]. Whereas, from the petroleum ether roots extract of *C. spinarum*, stigmasterol and campesterol were isolated by Karunakar et al. [108] using column chromatography. From the leaves of *C. carandas*, Begum et al. [41] and Bano et al. [95] isolated β-sitosterol-3-*O*-β-d-glucopyranoside (**55**).

### 5.5. Coumarins

Parveen et al. [72] reported one coumarin (6-hydroxy-7-methoxycoumarin) that was isolated from a methanol extract of the aerial parts of *C. opaca* (syn. *C. spinarum*). A coumarin-secoirdoid hybrid named carissaeduloside G was isolated from the root bark of *C. edulis* (syn. *C. spinarum*) [94].

### 5.6. Cardiac Glycosides

Six glycosides (carissaeduloside A (**24**), carissaeduloside B, carissaeduloside C, carissaeduloside D (**25**), carissaeduloside E, and carissaeduloside F) were isolated from the root bark of *C. edulis* (syn. *C. spinarum*) [94]. Out of all of these, carissaedulosides A (**24**) and carissaedulosides D (**25**) have been reported for their cytotoxic effects (Table 5) against cancer cell lines [94].

## 6. Pharmacological Profile

The traditional uses of *Carissa* species have inspired researchers to verify its utility through scientific pharmacological screening. Several crude extracts as well as bioactive constituents extracted from various plant parts have been evaluated for different biological activities, e.g., antioxidant, analgesic, anti-asthmatic, anticancer, anti-inflammatory, antidiabetic, antiulcer, anxiolytic, hepatoprotective, chemopreventive, hypotensive, and wound healing. Their medicinal potential has been observed in various animal models (in vitro as well as in vivo), scientifically proving the traditional utilisation of this plant.

### 6.1. Antioxidant Activity

Antioxidants are vital molecules that protect the body from harmful effects caused by free radicals. Their consumption reduces the mortality and morbidity caused by degenerative diseases [113,114]. Different assays successfully used by the researchers to analyse the antioxidant capacity in plants are the 2,2-azino-*bis*(3-ethylbenzthiazoline-6-sulphonic acid) free radical assay (ABTS), ferric reducing antioxidant power assay (FRAP), 2,2-diphenyl-β-picrylhydrazyl free radical assay (DPPH), phosphomolybdenum inhibition or total antioxidant capacity (TAC) assay, β-carotene bleaching, superoxide radical scavenging activity, lipid peroxidation assay, thiobarbituric assay, hydroxyl radical, and chelating power assay.

The antioxidant properties of the *Carissa* species have also been assessed by the methods mentioned above, and a detailed report of antioxidant potential of *Carissa* species is presented in Table 3. The most frequently used in vitro assays for *Carissa* species were the DPPH, ABTS, FRAP, and hydrogen peroxide scavenging activity assays, where ascorbic acid, rutin, and Trolox were used as the positive control. The DPPH assay showed that among all *Carissa* species, maximum antioxidant potential was observed in hydroethanolic leaves’ extract of *C. macrocarpa* (EC_50_ 26 µg/mL) [83,95], ethanolic leaves’ extract of *C. carandas* (IC_50_ 1.292 µg/mL) [44], and aqueous leaves’ extract of *C. spinarum* (syn. *C. opaca*) (EC_50_ 38 µg/mL) [74]. Similarly, the ABTS assay showed maximum antioxidant activity (EC_50_ 70 µg/mL) in the butanol fraction of methanol leaves’ extract of *C. spinarum* (syn. *C. opaca*) [74] and methanol extract of leaves of *C. carandas* (EC_50_ 1.75 µg/mL) [80]. Whereas the total antioxidant assay showed maximum antioxidant activity in the aqueous fraction of leaves of *C. spinarum* (syn. *C. opaca*) (EC_50_ 81 µg/mL) [74]. The thiobarbituric assay showed hydroethanolic stem extract of *C. macrocarpa* (EC_50_ 3.73 µg/mL) with good antioxidant activity [83], whereas hydrogen peroxide scavenging activity revealed better antioxidant activity in the hexane fraction of leaves’ extract of *C. spinarum* (syn. *C. opaca*) (EC_50_ 19 µg/mL) and the *n*-hexane extract of *C. carandas* (EC_50_ 1.802 µg/mL) [44,74]. In all other assays, such as scavenging ability of superoxide radicals, scavenging ability of hydroxyl radicals, and chelating power assays, different fractions of leaves and fruits of *C. spinarum* (syn. *C. opaca*) showed maximum antioxidant potential than the other species of *Carissa* (Table 3).

From Table 3, it is evident that *C. spinarum*, *C. carandas*, and *C. macrocarpa* have lower IC_50_ and EC_50_ values, which revealed their higher antioxidant potential. Whereas, in comparison to all species, *C. carandas* leaves have more antioxidant potential, followed by *C. macrocarpa* and *C. spinarum*. Various previous studies suggested that the fruits and leaves of *Carissa* species have significant antioxidant characteristics that can be useful as a potential preventive medication against free radicals-mediated disease as well as an antioxidant drug in the pharmaceutical and food industry [74,77,115].

Among all tested compounds for in vitro antioxidant activity, carissanol (**5**), carinol (**6**), and naringin (**19**), isolated from stem and leaves of *C. spinarum* and *C. carandas* by Wangteeraprasert et al. [23] and El-Desoky et al. [70] respectively, have shown good antioxidant activities. Similarly, olivil (**18**) (IC_50_ 18.1 µM) and carinol (**6**) (IC_50_ 20.2 µM) isolated from *C. spinarum* leaves revealed the highest antioxidant activity through the DPPH assay [19], whereas naringin (**19**) from *C. carandas* leaves also showed significant antioxidant potential (EC_50_ 11.2 µM) [67]. The literature shows that all studied *Carissa* species have strong in vitro antioxidant potential; however, in vivo studies need to validate the antioxidant potency of the genus.

### 6.2. Antimicrobial Activity

Various crude extracts and isolated compounds from different natural resources, especially from plants, have always been observed as a rich source of chemical compounds for controlling bacterial and fungal infections. Different assays that have been used in the literature for the screening of plant extracts for the antimicrobial potential are the Agar disk diffusion assay, Agar dilution assay, broth microdilution assay, and minimum inhibitory concentration (MIC) assay [116]. MIC was proposed to be the most prominent and accurate method to check microbe (bacteria/fungi) resistance to an antimicrobial drug or agent [117].

The overview of reported antimicrobial assays of leaves, stem, and root extracts of different *Carissa* species against Gram-negative and Gram-positive bacterial strains and some fungal human pathogens is presented in Table 4. Previous studies revealed good antibacterial activity (in vitro) of all plant parts (leaves, fruits, and roots) of *Carissa* species against human pathogens such as *Bacillus subtilis, Pseudomonas aeruginosa, Enterococcus faecalis, Escherichia coli, Klebsiella pneumoniae* [35,46,88], *Salmonella typhi* [76]*, Candida albicans* [7,22,34,89], and *Streptococcus pyogenes* [22,34]. The root extract of *C. spinarum* was found most active against *P. aeruginosa* at MIC of 8.0 µg/mL [35]*,* and *Staphylococcus aureus* at MIC of 312 µg/mL [118]. In contrast, fruits extract of *C. carandas* showed good activity against *K. pneumoniae* and *S. aureus,* with the same MIC of 0.3125 mg/mL [87]. Similarly, *n*-butanol fraction from the root and leaves’ extracts of *C. macrocarpa* (syn. *C. grandiflora*) showed maximum antibacterial activity against *S. epidermidis* with MIC of 0.24 (roots) and 0.56 mg/mL (leaves), and *S. aureus* with MIC of 0.82 (roots) and 0.67 mg/mL (leaves) [89].

The essential oil of the stem of *C. macrocarpa* has also shown good antimicrobial activity against *Salmonella enterica* and *B. subtilis* (MIC 0.46 mg/mL) [7]. The overall comparative study shows that *C. carandas* and *C. spinarum* are most effective against bacterial pathogens. Whereas, against fungal pathogens (*Alternaria solani, C. albicans, Aspergillus flavus,* and *Penicillium monotricale*), only the ethyl acetate fraction from *C. spinarum* root extract (syn. *C. opaca)* (MIC 7.8 µg/mL) and essential oil from the fruits of *C. macrocarpa* (MIC 0.46 mg/mL) have been screened by Awasthi et al. [34] and Souilem et al. [7]. No data are available on the antifungal activity of other *Carissa* species.

Additionally, ursolic acid (**2**) and 3β-hydroxyolean-11-en-28,13β-olide (**14**) (from *C. macrocarpa* leaves and fruits, respectively) [8], and dehydrocarissone (**15**), carindone (from the wood of *C. spinarum*) [1], 2-hydroxyacetophenone (**13**), carinol (**6**), and carissone (**3**) (from the root of *C. spinarum*) [39] have also been reported for their antibacterial activity. The compound, 3β-hydroxyolean-11-en-28,13β-olide (**14**), was found most active towards *S. aureus, E. coli,* and *P. aeruginosa* with MIC value of 0.06 mg/mL [8]. The above literature with quantitative data in terms of low MIC values supported the broad-spectrum antibacterial potential of *Carissa* species; however, the antifungal potential of *Carissa* species needs to be explored.

### 6.3. Anticancer Activity

The anticancer potential of crude extracts and purified constituents of *Carissa* species has been observed by various researchers on different cell lines, such as human cervical (Hela) cell line, breast cancer (MCF-7), hepatocellular carcinoma (HepG2), bone sarcoma (MG-63), ovarian cell (OVCAR-5), prostate cell (PC-3), human lung cancer (A549), human leukaemia (HL-60), colon cancer (SW480), and normal human (WI38) cell lines (Table 5). The root extract of *C. spinarum* showed significant anticancer activity (IC_50_ 34.58 µg/mL for leukaemia HL-60; GI_50_ 18.1 µg/mL for breast cancer) [93,98]. Likewise, *n*-hexane unripe fruit extract of *C. carandas* also showed significant anticancer activity (EC_50_ 2.492 µg/mL) for lung cancer cell lines [97,115]. Simultaneously, methanol fruit extract of *C. carandas* was observed as the most active against breast cancer, with an IC_50_ of 56.72 µg/mL [90].

Carandinol (**17**), isolated from *C. carandas* leaves, showed better anticancer activity against cervical, prostate, and normal mouse fibroblast cancer cell lines. Carandinol (**17**) from *C. carandas* also showed prominent anticancer properties towards human cervical cancer, with an IC_50_ of 6.87 µM [41]. Sarhamnoloside (**22**), a constituent from *C. spinarum* root bark (syn. *C. edulis*), showed strong cytotoxic potency against human leukaemia cell lines (IC_50_ 0.029 µM), lung cancer (IC_50_ 0.023 µM), breast cancer (IC_50_ 0.025 µM), and colon cancer (IC_50_ 0.137 µM) [94]. Similarly, carissaeduloside A (**24**), D (**25**), and J (**26**) isolated from *C. spinarum* (syn*. C. edulis*) were screened for cytotoxic potential using human leukaemia, lung cancer, breast cancer, and colon cancer cell lines. Among these, carissaeduloside J (**26**) was observed with the best anticancer activity against lung cancer (3.87 µM) and breast cancer (9.231 µM). The anticancer activity of other compounds is shown in Table 5. Among all the tested extracts, hexane extract of unripe fruits of *C. carandas* showed the highest anticancer activity against lung cancer [97].

Numerous researchers have reported the anticancer potential of *Carissa* species against various cell lines. However, further investigation needs to trace the mechanism of action of extracts/compounds against cancer cell lines.

### 6.4. Antiplasmoidal and Antimalarial Activity

*C. spinarum* and C. *carandas* are the only two species that have been screened for antimalarial and antiplasmoidal activities. Kebenei et al. [118] revealed that the methanolic extract of the *C*. *spinarum* root bark (syn. C. edulis) exhibited significant antiplasmoidal activity (IC_50_ 1.95 mg/mL) against D6 strains (chloroquine-sensitive) of *Plasmodium falciparum* parasite, which could be due to the presence of nortrachelogenin (**23**) (IC_50_ 14.50 µg/mL). According to the authors, the crude extract of the root bark of *C. spinarum* was a good and easily available source for the development of an antimalarial drug [119,120]. Similarly, Bapna et al. [120], through their in vitro study on *P. falciparum* 3D7 strains, stated higher antimicrobial potential of the methanol extracts of leaves, stem, bark, and fruits from *C. carandas* (IC_50_ 13.57–69.63 μg/mL), in contrast to aqueous extracts (IC_50_ 41.52–100 μg/mL). The authors used the 3-[4,5-dimethylthiazol-2-yl]-2,5-diphenyltetrazolium bromide (MTT) assay to analyse host cell cytotoxicity on the Madin-Darby Canine Kidney (MDCK) cell line and observed no cytotoxicity in the maximum tested dose.

Gebrehiwot et al. [120] screened the hydro-alcoholic and chloroform root extracts of *C. spinarum* for antiplasmoidal activity. They observed that both extracts exhibited significant activity (*p* < 0.05) at 400 mg/kg against *P. berghei* in Swiss albino mice (43.23% ± 0.66% parasitic suppression at day hour with hydroalcoholic extract and 51.64% ± 2.13% inhibition with chloroform extract), where chloroquine phosphate was used as a positive control (51.82% ± 0.72% parasitic suppression). The authors also observed that plant extract-treated mice did not exhibit any acute toxic effects up to 2000 mg/kg.

These data support the ethnopharmacological information about the antimalarial activity of *Carissa* species. The in vivo assays revealed the antimalarial property at 400 mg/kg of the extract, and according to Gertsch [121], for the in vivo assay, a significant concentration should be >200 μg/mL. Therefore, more investigations are required to develop clinical trials that can prove the efficacy of *C. spinarum* and *C. carandas* in humans to treat malaria.

### 6.5. Antiviral Activity

Only two species of *Carissa* viz., *C. spinarum* (root bark) and *C. carandas* (fruits), have been explored for antiviral activity by Tolo et al. [106,122] and Arif et al. [12], respectively. Tolo et al. [122] observed significant in vitro antiviral activity (*p* < 0.05) of *C. spinarum* (syn. *C. edulis*) root extract at 1 mg/mL against herpes simplex viruses HSV-I (EC_50_ 15.1 ± 0.57 µg/mL), HSV-II (EC_50_ 6.9 ± 1.27 µg/mL), HSV-I AP^r^ (EC_50_ 8.1 ± 1.56 µg/mL), and HSV-I TK^-^ (EC_50_ 11.1 ± 5.66 µg/mL), in comparison to the significant antiviral activity of Acyclovir (positive control) against HSV-I (EC_50_ 0.91 ± 0.46 µg/mL), HSV-II (EC_50_ 0.87 ± 0.44 µg/mL), HSV-I AP^r^ (EC_50_ > 5.0 µg/mL), and HSV-I TK^-^ (EC_50_ > 5.0 µg/mL). According to Tolo et al. [123], 250 mg/kg roots’ extract of *C. spinarum* (aqueous) possessed good anti-herpes simplex virus activity in comparison to acyclovir (5 mg/kg); as a result, it may be used for developing an effective treatment against HSV infections.

Tolo et al. [106] also screened various phytocompounds, such as lupeol (**1**), oleuropein (**20**), carissol (**12**), and α-amyrin (**21**), from the *C. spinarum* root bark for antiviral activity (in vitro) against Herpes simplex virus strains 1 (7401H HSV-1, TK^-^ B2006 HSV-1, AP^r^ 7401H HSV-1), up to 1 mg/mL concentration. The study revealed a significant activity (*p* < 0.05) of Lupeol (**1**) for both sensitive and resistant strains (EC_50_ 2.98 μg/mL for 7401H HSV-1; EC_50_ 3.66 μg/mL for AP^r^ 7401H HSV-1; EC_50_ 4.2 μg/mL for TK^-^B2006 HSV-1), with a high therapeutic index (TI > 38) similar to the positive control (EC_50_ 0.45 μg/mL for 7401H HSV-1; EC_50_ > 10 μg/mL for AP^r^ 7401H HSV-1; EC_50_ > 10 μg/mL for TK^-^B2006 HSV-1). According to the authors, the compound at 10 μg/mL was found as virucidal, as evident in E6 cells by 98.3% (in vitro). Arif et al. [12] explored the effectiveness of *C. carandas* fruits’ ethanolic extract (6, 3, and 12 μg/mL) for antiviral activity against Poliovirus HIV-1, Sindbis virus, and Herpes simplex virus, respectively.

Whereas, for the in vivo study, *C. spinarum* (syn. *C. edulis*) at 250 mg/kg showed significant antiviral activity (delayed infection by two days) against 7401H HSV-1-infected Balb/C mice (female), and similar results were obtained with the 5 mg/kg positive control. At 250 mg/kg, mice showed no signs of toxic effects.

From the above studies, it has been made clear that *Carissa* species have significant activity towards different Herpes simplex viral strains due to the presence of lupeol (**1**). The members of this genus can also be used to formulate effective drugs against other types of viruses.

### 6.6. Anticonvulsant Activity

Anticonvulsant activity was only observed with *C. spinarum* and *C. carandas*. All studies suggested biologically active constituents in the root bark of *C. spinarum* and *C. carandas* that have anticonvulsant activity. The anticonvulsant activity of different fractions (aqueous, *n*-butanol, and ethyl acetate) obtained from hydro-alcoholic (60% *v*/*v*) root bark extract of *C. spinarum* (syn. *C. edulis*) was observed against maximal electroshock (MES) in chicks, pentylenetetrazole (PTZ), and 4-aminopyridine-induced seizure (90 and 15 mg/kg, respectively) in mice at dose levels between 1.25 and 10 mg/kg by Jamilu et al. [124]. The researchers observed 80% anticonvulsant activity in the aqueous fraction (10 mg/kg) in PTZ-induced seizures as compared to valproate (200 mg/kg), a standard antiepileptic drug that showed 100% protection. Whereas, ethyl acetate and *n*-butanol fractions also showed protection, but that was not dose-dependent. In MES-induced seizures in chicks, 30% protection was observed with an ethyl acetate fraction at 2.5 mg/kg, and according to the authors, the entire fractions reduced the mortality rate in a dose-independent manner. Thus, it was observed that all used fractions of *C. spinarum* have anticonvulsant activity, particularly against MET- and PTZ-induced seizures.

Yau et al. [5] investigated the LD_50_ of *C. spinarum* (syn. *C. edulis*) root bark extract on convulsions induced by pentylenetetrazole (PTZ) and maximal electroshock (MES) (in mice and chicks, respectively) through oral and intraperitoneal administration. The LD_50_ values of 282.8 (intraperitoneal) and 5000 mg/kg (oral) were observed for *C. edulis* root extract. Further, the extract exhibited 40% and 20% inhibition of convulsions in mice induced by PRZ at 20 and 5 kg/mL respectively, as compared to benzodiazepine (100% inhibition). On MES-induced convulsions in chicks, root extract showed 90% inhibition compared to 100% protection with 20 mg/kg of phenytoin. In the year 2009, the anticonvulsant effect of a root bark ethanolic extract from *C. carandas* was studied by Hegde et al. [21] on electrically, picrotoxin, chemically, *N*-methyl-dl-aspartic acid, pentylenetetrazole, and bicuculline-induced seizures in a mouse model. The study showed that ethanolic extract (100–400 mg/kg) reduced (*p* < 0.001) the duration of seizures induced by MES. At 100, 200, and 400 mg/kg of extract, 25%, 50%, and 62.5% inhibition of seizures was observed. Whereas in another case, the seizures were induced with pentylenetetrazole, and similar doses showed significant protection and delayed progression of tonic seizures formed by *N*-methyl-dl-aspartic acid and picrotoxin. On the contrary, the extract showed an insignificant effect against bicuculline-produced seizures. From the above information, it is clear that *C. spinarum* and *C. carandas* roots exerted significant anticonvulsant effects in in vivo studies, and therefore, both species can be exploited for the treatment of epilepsy.

### 6.7. Antinociceptive Activity

The methanolic extract from *C. carandas* leaves (50, 100, 200, and 400 mg/kg) exhibited significant antinociceptive activity using the Swiss albino mice gastric pain model (acetic acid as an inducer). It decreased the number of writhings as compared to the antinociceptive standard drug aspirin (at 200 and 400 mg/kg) [123]. In another study, the methanolic extract from *C. spinarum* (syn. *C. edulis*) leaves reduced 47.04–47.19% and 38.96–89.26% of pain in rats in the early and late phase respectively, at 100 mg/kg (47.04%) and 150 mg/kg (47.19%) body weight. However, methanol root bark extracts reduced it by 21.5–41.89% (early phase) and 21.4–90.62% (late phase) in comparison to diclofenac (15 mg/kg, standard drug), which reduced the pain by 27.37–34.9% and 88.24–90.28% in the early and late phase, respectively [125]. Mworia et al. [126] observed a 73.77% and 86.89% reduction in acetic acid-associated pain in a mouse model by using acetone leaves’ extract of *C. spinarum* (50 and 100 mg/kg body weight, respectively), in comparison to diclofenac sodium (70.49% at 15 mg/kg). Similarly, Parvin [20] also observed significant (*p* < 0.01) antinociceptive activity in Swiss albino mice with *C. carandas* (leaves’ methanolic extract) at 200 and 400 mg/kg body weight, as compared to the standard drug (diclofenac, 1 mg/kg). Traditionally, *Carissa* plants have been reported in different forms such as decoction, infusion, etc., to treat fever and pain, and these in vivo studies provide evidence for the antinociceptive effect of the *Carissa* species.

### 6.8. Antidiabetic Activity

The ethanolic leaves’ extract from *C. spinarum* (syn*. C. edulis*) (2 g/kg) was observed with significant antidiabetic activity in comparison with the reference drugs (metformin (500 mg/kg) and 3 mg/kg of glibenclamide) in diabetic adult male albino rats [127]. Swami et al. [128] observed that an aqueous extract from *C. carandas* at 500 and 1000 mg/kg showed antidiabetic activity (*p* < 0.05) by decreasing the blood glucose levels in diabetic Wistar rats (alloxan-induced model). Furthermore, the authors observed that methanol extracts also lowered the elevated levels of blood glucose significantly (*p* < 0.001) after 24 h at 400 mg/kg orally, as compared to the control. This was attributed to the total polyphenols and total flavonoids content of the plant. Itankar et al. [129] observed a decrease in the level of blood glucose (48% and 64.5%) in Wistar rats after using 400 mg/kg per oral methanol extract of unripe *C. carandas* fruit extract and its fraction (ethyl acetate), respectively. The authors also revealed the higher antidiabetic potential in the case of ethyl acetate fraction, in contrast to methanol extract. They concluded that fractionation increased polymerisation/segregation of biologically active metabolites.

On the other hand, Madhuri and Neelagund [130] stated that the aqueous extract from *C. carandas* fruits showed potent inhibition of β-glucosidase activity between 1.25 and 10 mg/mL concentration.

These data also support the traditional and practical information about the antidiabetic activity of *Carissa* species. Leaves and fruit extracts of *C. spinarum* and *C. carandas* significantly inhibit the blood glucose level and can be used to isolate bioactive constituents in drug preparations for human welfare.

### 6.9. Antipyretic Activity

Various researchers observed the antipyretic activity of leaves and roots of the *Carissa* genus and therefore validated its folk use in the treatment of pain and fever. Hegde and Joshi [57] observed that the ethanolic root extract from *C. spinarum* (100, 200, and 400 mg/kg) reduced the body temperature in Wistar rats (Brewer’s yeast-induced pyrexia), and therefore stated the significant activity (*p* < 0.05) of ethanolic extract. Garg et al. [131] also showed the significant antipyretic activity (*p* < 0.01) of aqueous extract of *C. carandas* leaves at all dose levels (100, 200, and 400 mg/kg) compared to 200 mg/kg of paracetamol. Hati et al. [132] and Bhaskar and Balakrishnan [133] also stated that methanolic extracts of *C. carandas* leaves and roots at 100 and 200 mg/kg reduced (*p* < 0.01) pyrexia in the albino rats in a dose-dependent manner. According to Gitahi [134], the leaves and the root bark extract (dichloromethane:methanol) of *C. spinarum* (syn. *C. edulis*) have profound antipyretic activities compared to the drug (100 mg/kg, aspirin) at a concentration of 50, 100, and 150 mg/kg. On the other hand, Allam et al. [135] also showed the highest antipyretic potential of the crude methanolic extract and butanol fraction of the aerial parts (leaves and stem) of *C. macrocarpa* at 100 mg/kg in a yeast-induced hyperpyrexia model, in male albino rats. Acetylsalicylic acid (100 mg/kg) was used as a standard drug.

The antipyretic data show that the authors have used a high concentration of extracts for the experiments; therefore, further in vivo studies need to validate the traditional use of different parts of the genus *Carissa.*

### 6.10. Anti-Inflammatory Activity

The different plant parts of the genus *Carissa* such as roots, stem, and leaves have been screened for anti-inflammatory activity by various researchers. Bhaskar and Balakrishnan [133] screened the root extracts from *C. carandas* (ethanol and aqueous) for anti-inflammatory activity. It was found that both the extracts significantly reduced the formation of oedema induced by carrageenan after 2 h. Anupama et al. [42] reported the anti-inflammatory activity of methanol extract from *C. carandas* fruits using a carrageenan-induced hind paw oedema model. The extract at all dose levels (100, 200, and 400 mg/kg) showed significant activity when administered orally to the experimental rats as compared with 50 mg/kg of indomethacin. Hati et al. [132] studied the effect of *C. carandas* leaf methanolic extract (200 mg/kg) against dextran, histamine, and carrageenan-induced paw oedema, and observed 71.90%, 72.10%, and 71.80% inhibition respectively, at the end of 3 h.

Beck and Namdeo [136] studied the anti-inflammatory activity of different extracts from *C*. *spinarum* leaves (petroleum ether, chloroform, alcoholic, and aqueous) using a formalin-induced rat paw oedema model in rats. The extracts at 200 mg/kg exhibited anti-inflammatory activity (*p* < 0.01) as compared to the control metamizole (20 mg/kg). Among the extracts, the aqueous extract was found to be the most potent as compared to other extracts tested. Yau et al. [137] evaluated the anti-inflammatory effects of a residual aqueous fraction from the *C. spinarum* (syn. *C. edulis*) root bark ethanolic extract using a carrageenan-induced paw oedema model in rats. It was found that the residual fraction at 600 mg/kg and standard ketoprofen at 10 mg/kg showed a decrease (*p* < 0.05) in oedema up to 4 h. According to Woode et al. [138], anti-inflammatory activities (in vivo) in the alcoholic extract of *C. spinarum* (syn. *C. edulis*) roots could be due to the presence of antioxidants.

Saher et al. [139] observed the anti-inflammatory activity of *C. carandas* fruits (immature, mature, and ripe) using carrageenan-induced paw oedema and cotton pellets-induced granuloma in Wistar rats. The highest percentage inhibition was observed with mature fruit extract (200 mg/kg) at 3 h. Whereas, at a 100 mg/kg dosage, immature fruit extract exhibited 68% (highest) inhibition in the carrageenan-induced model compared to mature and ripe fruit extracts after 3 h. On the other hand, in the cotton pellet method, less than 50% inhibition of granuloma was found with the immature (21.93%), ripe (20.14%), and mature (24.91%) fruit extracts compared to standard diclofenac sodium (45%). Allam et al. [135] screened the ethyl acetate and dichloromethane fractions from crude methanolic extract (leaves and stem) of *C. macrocarpa* aerial parts for anti-inflammatory activity and observed maximum activity at 100 mg/kg, with 43% and 41% inhibition respectively, in comparison with the standard drug indomethacin (47% at 10 mg/kg).

In case of isolated compounds, the anti-inflammatory potential of naringin (**19**) from *C. carandas* leaves at 50 mg/kg showed potent inhibition (59.24%) of inflammation when compared with the control (48.10% with 20 mg/kg of indomethacin) [70].

On the other hand, carisssone (**3**) and scopoletin (**8**), purified from *C. carandas* roots, also showed inhibition of nitric oxide (NO) production (IC_50_ 20.1 ± 2.99 and 24.6 ± 1.36 µg/mL, respectively) as compared to the standard inhibitor of NO (IC_50_ 19.82 ± 1.64 µg/mL) with no adverse effect on cell viability [110]. It is clear from the above data and traditional information that different plant parts of *C. spinarum* and *C. carandas* were found to be anti-inflammatory, and their effect is related to their phytoconstituents, such as carissone (**3**) and naringin (**19**). However, the mechanistic basis of these extracts/compounds remains obscure.

### 6.11. Hepatoprotective Activity

Bhaskar and Balakrishnan [140] studied the hepatoprotective effects of the *C. carandas* root extracts (aqueous and ethanolic) in rats. The authors observed that both extracts at doses of 200 and 100 mg/kg exhibited significant hepatoprotection (*p* < 0.05) by reducing lipid peroxidation, serum transaminase, alkaline phosphate, and bilirubin, while elevating the serum as well as liver glutathione levels (*p* < 0.01) as compared to standard aspirin (150 mg/kg). Subsequently, Hegde and Joshi analysed the hepatoprotective effect of *C. spinarum* roots’ ethanolic extract in rats. The authors stated that pre-treatment (orally) with extract (100, 200, and 400 mg/kg) alleviated (*p* < 0.01) the hepatotoxicity induced by CCl_4_ and paracetamol in a dose-dependent manner [141]. According to the authors, root extracts reduced the hepatotoxicity in rats by decreasing lipid peroxidation and bilirubin and enhancing the level of protein, glutathione, uric acid, catalase, and superoxide dismutase.

Similarly, Sahreen et al., also observed the hepatoprotective activity of methanol extract of *C. spinarum* leaves (syn. *C. opaca*) in Male Sprague-Dawley rats [58]. The authors stated that the extract at 200 mg/kg significantly reduced CCl_4_-induced hepatotoxicity in rats as compared to the positive control, 50 mg/kg of silymarin. It was concluded that the activity in leaves could be due to its antioxidant activity and membrane-stabilizing potential.

El-Desoky et al. evaluated the defatted aqueous methanol leaves’ extract (500 mg/kg) of *C. carandas* for its hepatoprotective effects and compared it with the drug silymarin (100 mg/kg) [70]. The authors observed that the extract significantly (*p* < 0.01) reversed elevated serum liver marker enzymes, reduced malondialdehyde (MDA), and subsequently increased glutathione (GSH) content in liver homogenate.

The crude extracts, their fraction, and pure compounds isolated from the plants have been proven as a very effective drug for liver disease. These extracts possessed sufficient efficacy to treat severe liver disease caused by toxic chemicals, viruses, and excess alcohol. Thus, from the above findings, it can be stated that *Carissa* species such as *C. spinarum* and *C. carandas* are promising hepatoprotective agents, validating the assertion of traditional healers.

### 6.12. Antiarthritic Activity

Hegde et al. [84] observed the significant (*p* < 0.05) and dose-dependent anti-arthritic activity of ethanolic root extracts of *C. spinarum* (100, 200, and 400 mg/kg, p.o.) and phenylbutazone (100 mg/kg, i.m.) in Freund’s adjuvant-induced polyarthritis model in rats. Dar et al. [6] also studied the antiarthritic activity of ethanolic leaves’ extract from *C. carandas* (200 and 400 mg/kg) in arthritis model rats (adjuvant-induced) and observed that the ethanol extract showed a reduction (*p* < 0.01) in paw volume when compared with aspirin (50 mg/kg). According to the authors, this property is attributed to the synergistic potential of phytoconstituents.

The above-discussed data suggested the significant antiarthritic potential of leaves and root extracts of *C.* *spinarum* and *C. carandas,* which may be possibly due to lanost-5-en-3β-ol-21-oic acid (**27**). However, the role of other phytocompounds of the genus needs to be explored.

### 6.13. Adaptogenic Activity

Arif et al. [87,109] screened the crude ethanolic extract and a lanostane triterpenoid, lanost-5-en-3β-ol-21-oic acid (**27**), from the *C. carandas* fruit ethanolic extract (of 200, 100, and 10 mg/kg/day), and it was evaluated for the adaptogenic activity using anoxia stress tolerance, swimming endurance, and immunosuppression induced by cyclophosphamide (experimental mice) models. Aspirin (25 mg/kg) was used as the standard drug. The authors observed the great role of crude extract and lanostane triterpenoid in cyclophosphamide-treated mice with an increase in swimming endurance, anoxia stress tolerance, and normalcy of parameters such as Hb, affected organ, RBC, WBC, and body weight (*p* < 0.05 and *p* < 0.01, respectively). Therefore, the authors revealed the significant adaptogenic activity of lanostane triterpenoid and the crude extract as well.

### 6.14. Effect on the Cardiovascular System and Cardioprotective Activity

The cardioprotective activity and the effect of *C. spinarum* aerial parts on the cardiovascular system were evaluated by Al-Youssef and Hassan [69] and Sahreen et al. [142]. The cardiovascular effect of different extracts from *C. spinarum* (syn. *C. edulis*) aerial parts was evaluated in Wistar rats. The authors observed that ethyl acetate, petroleum ether, and aqueous extracts (0.05 g/kg) showed an observable decline in arterial pressure (27, 27.2, and 9.1 mmHg, respectively), whereas the extracts at 0.1 g/kg exhibited a 34.5, 36.3, and 32.7 mmHg decline in blood pressure, respectively [64].

Subsequently, Sahreen et al. [143] observed the cardioprotective potential of different fractions (methanol, *n*-hexane, ethyl acetate) from *C. spinarum* (syn. *C. opaca*) leaf extracts in male Sprague Dawley rats whose cardiac function was altered by treating with carbon tetrachloride (CCl_4_). The authors observed that all tested fractions showed protective effects against CCl_4_ intoxication, by normalizing the altered cardiac function and antioxidant enzymes. In addition to these, DNA damage and histopathological abnormalities were also restored by various fractions. However, further studies are required to validate the claim of the genus *Carissa* for their action against cardiovascular activity. Additionally, further studies need to include the mechanism of action of extracts for their cardioprotective potential using different model systems [143].

### 6.15. Anthelmintic Activity

Only two species of *Carissa* (*C. spinarum* and *C. carandas*) have been screened for anthelmintic activity. The first study on the anthelmintic activity of the *C. carandas* root bark was performed by John et al. [142]. According to the researchers, 50 mg/mL of the methanol extract caused paralysis on Indian earthworm, followed by the death of the worm, hence revealing anthelmintic activity of plant roots comparable with that of the standard drug albendazole (10 mg/mL). In another study, Harwansh et al. [37] stated that methanolic and chloroform extract of *C. spinarum* were found equally potent at 100 mg/mL, as compared to piperazine citrate (10 mg/mL), which resulted in paralysis that led to the death of *Pheretima posthuma*. Mishra et al. [144] also observed in vitro anthelmintic activity in unripe fruits of *C. carandas* at doses of 50, 100, and 150 mg/mL on Indian earthworms, and detected a short time of paralysis (56.35, 40, and 22.35 min) followed by the worms’ death at 150 mg/mL of ethanolic, chloroform, and petroleum ether extracts respectively, when compared to piperazine citrate (15 mg/mL). These observations indicated that ethanolic extract took a shorter duration to cause paralysis when compared with unripe fruit extract. Similarly, Parvin [20] studied the anthelmintic activity of fresh juice of *C. carandas* leaves (25, 50, and 100 mg/mL) on earthworms by observing their paralysis and death time. The authors observed the paralysis and death time (minimum to maximum) in the range of approximately 4 to 7 min respectively, compared to the standard drug albendazole (100 mg/mL; 3–10 min). The authors stated that the fresh juice of leaves showed potent anthelmintic activity in earthworms.

### 6.16. Antiemetic Activity

Mohtasheemul et al. [145] also studied and compared the antiemetic activity of *C. carandas* fruits and the reference drug domperidone (100 mg/kg) on a chick emetic model. Their results showed that the extract decreased the number of copper-sulphate pentahydrate-induced retches (50 mg/kg body weight, orally) in chicks. However, other members of the genus *Carissa* also need to be investigated for their anthelmintic and antiemetic potential. Additionally, studies need to be designed based on the effect of active compounds of plant extracts on the death of pathogenic worms.

### 6.17. Neuropharmacological and Diuretic Activity

Various researchers observed the neuropharmacological and diuretic activity of leaves and root bark extracts. Saha et al., studied the methanol extract of *C. carandas* to evaluate neuropharmacological and diuretic activities on male albino rats [146]. Their findings revealed significant (*p* < 0.01) neuropharmacological activity of the extract (250 and 500 mg/kg). The diuretic activity of the extract was evident from the 1.46 to 1.43 reduction in the ratio of Na^+^/K^+^ excretion at 200 and 400 mg/kg respectively, when compared to the standard diuretic furosemide (1.48, 0.5 mg/kg).

The neuroprotective effect of the aqueous extract on the *C. spinarum* (syn. *C. edulis*) leaves was also evaluated by Yadang et al. [147] using the novel object recognition, T-maze methods in mice to identify memory, learning, open-field locomotion test, along with brain acetylcholinesterase enzyme (AChE) activity. In addition to these, the authors also examined oxidative stress through different parameters such as malondialdehyde (MDA) level, glutathione, and catalase activity. The results showed that at different dose levels, such as 62.8, 143, 314, and 628 mg/kg, plant extract (orally administrated) increased the memory, object recognition, and also improved the locomotion of mice. Whereas Scopolamine (1 mg/kg body weight) administration for seven days of treatment showed a decrease in learning and memory enhancement in mice. On the other hand, mice with aqueous extract decreased the AChE activity (2.55 ± 0:10 mol/min/g) and brain oxidative stress. The authors concluded that by reducing AChE activity, aqueous extract enhanced the memory of mice.

Nedi et al. [148] observed the significant (*p* < 0.01) diuretic property of an extract from the *C. spinarum* (syn. *C. edulis*) root bark at 1000 mg/kg, and wood maceration (50 mg/kg). According to the authors, the plant extract contains compounds that mediated the diuretic effect by significantly increasing the volume of urine and also enhanced the number of electrolytes, K^+^, Na^+^, and Cl^-^ ions. AI-Youssef and Hassan [68] compared the diuretic potential of different extracts (petroleum ether, ethyl acetate, chloroform, and aqueous) from *C. spinarum* aerial parts at a dose of 1 g/kg, in contrast to the control (normal saline). Their result showed that petroleum ether and ethyl acetate extract (1 g/kg) slightly affected urine output, with 9.1% and 12.7% respectively, while chloroform and aqueous extracts at the same dose significantly increased urine output by 54.5% and 45.4%, respectively. Kebamo et al. [149] observed the significant diuretic activity of an aqueous fraction from *C. spinarum* (syn*. C. edulis*) root bark methanolic extract at doses of 50, 500, and 1000 mg/kg in normal Wistar rats. On the other hand, *n*-butanol and petroleum ether fractions were devoid of activity as compared with standard hydrochlorothiazide (10 mg/kg).

The concentration of extracts used in the above-discussed studies was relatively high. Furthermore, in vivo studies are required to validate the utilisation of *Carissa* species for the neuropharmacological and diuretic properties.

### 6.18. Wound Healing Activity and Toxicological Study

Sanwal and Chaudhary [150] applied cold macerated 1% and 2.5% methanolic extract of *C. spinarum* root extract against a burn wound mice model and observed wound contraction and epithelisation, therefore proving the significant wound healing potential of the root extract. Subsequently, the in vivo toxicity (acute as well as subacute) of the root extract of *C. spinarum* in Swiss albino mice was evaluated by Gebrehiwot [151]. According to the researchers, the hydro-methanolic and chloroform extracts at a 5000 mg/kg dose did not produce significant physical and behaviour changes, and no death was recorded. Whereas, in sub-acute toxicity studies, the extracts showed an insignificant change (*p* > 0.05) of haematological and physical parameters in the treated groups when associated with the control groups. Shamim also studied the acute, subacute, and sub-chronic toxicological studies of the ethanolic extracts of *C. carandas* leaves. The authors reported that the extracts at 1750 and 5000 mg/kg did not exhibit any mortality in the acute toxicity evaluation, whereas subacute toxicity exhibited no signs of toxicity and mortality in the treated group, contrary to the control ones at 5000 mg/kg [152]. On the other hand, chronic toxicity (5000 mg/kg) showed some changes in the histological parameters. The ethanolic extract of *C. spinarum* roots at 2000 mg/kg exhibited no toxicity or behavioural changes in Wistar albino rats during 14 days of treatment [57]. They have an important regulatory role and are therefore seen as therapeutic goals of *Carissa* species to control the wound healing processes in the future.

The safety evaluation of *C. carandas* extract (5000 mg/kg) was evaluated by Bhaskar and Balakrishnan [133]. According to the authors, this dose was tolerated by rats, and no adverse symptoms or deaths have been observed in acute toxicity investigation. However, an oral LD_50_ of the extract was found unascertainable in rats (>5000 mg/kg body weight). According to the authors, the plant extract is considered non-toxic if oral LD_50_ values are higher than 4 g/kg [153,154]. Recently, an in vivo acute toxicity assay of *C. spinarum* (syn. *C. edulis*) extracts (methanol and methanol:water) at doses of 50–2000 mg/kg showed that there was no behavioural change or death observed during seven days of treatment [91]. According to the authors, both extracts were found safe at doses of up to 2000 mg/kg body weight in mice. Although the literature proved the wound healing effect of the root extract of *C. spinarum,* still more investigations are required. Toxicity studies that were performed on *Carissa* species also showed non-toxicity of *C. spinarum* and *C. carandas* extracts up to 5000 mg/kg. Dossou-Yovo et al. [155] also proved the safety of the *C. spinarum* roots’ hydroalcoholic extract at 500, 1000, and 5000 mg/kg by conducting acute and subacute oral toxicity on Wistar rats through the oral route.

## 7. Conclusions and Summary

*Carissa* genus is a key contributor of valuable phytoconstituents, and various *Carissa* species have been screened for their nutrients, bioactive constituents, and for pharmaceutical aspects, which are important for maintaining good health. The present review is focused on the research carried out on various aspects of only four species of *Carissa* (*C. spinarum, C. carandas, C. macrocarpa,* and *C. bispinosa*). The other species reported in the literature, such as *C. opaca*, *C. edulis*, *C. lanceolata*, *C. congesta,* and *C. grandiflora,* are the synonyms of *C. spinarum* and *C. macrocarpa*, respectively. A total of 121 compounds (35 polyphenols (phenolic acids and flavonoids), 30 lignans, 41 terpenoids, 7 steroids, 2 coumarins, and 6 cardiac glycosides) have been extracted from *C. spinarum, C. carandas,* and *C. macrocarpa*. No report is available on the isolation of chemical constituents from *C. bispinosa.* Among all these compounds, only a few compounds have been screened/tested for different biological activities. For example, lupeol (**1**), oleuropein (**20**), carissol (**12**), and α-amyrin (**21**), extracted from *C. spinarum,* have been tested against herpes simplex viruses (I, II, III), naringin (**19**), carisssone (**3**), and scopoletin (**8**), isolated from *C. carandas,* have been checked only for anti-inflammatory activity, and Carandinol (**17**), sarhamnoloside (**22**), and carissaeduloside A (**24**), D (**25**), and J (**26**), from *C. carandas* and *C. spinarum* respectively, were explored for anticancer activity. Similarly, carissanol (**5**) and naringin (**19**), isolated from *C. spinarum* and *C. carandas,* and olivil (**18**) and carinol (**6**) isolated from *C. spinarum* have shown good antioxidant activities. Besides these, 3β-hydroxyolean-11-en-28,13β-oilde (**14**) and ursolic acid (**2**), isolated from *C. macrocarpa,* and carinol (**6**) and carissone (**3**) from *C. spinarum* have been evaluated for antimicrobial activities.

**Figure 4:**
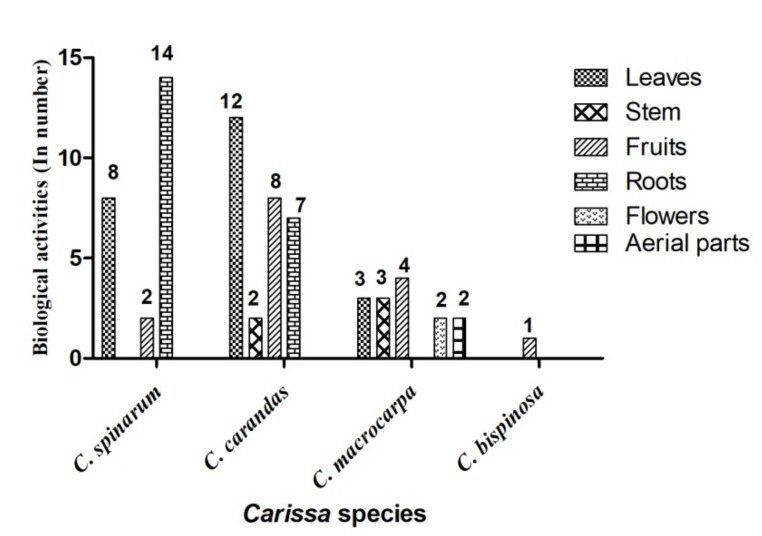
Ratio of biological activities screened during the analysis of the literature from *C. spinarum*, *C. carandas*, *C. macrocarpa*, and *C. bispinosa.*

Among all the *Carissa* species, most of the pharmacological activities were tested on *C. spinarum* and *C. carandas,* whereas *C. macrocarpa* was used to evaluate antioxidant, antimicrobial, anticancer, antipyretic, and anti-inflammatory activities. The most extensively used parts from various reported activities were the roots and leaves of *C. spinarum* and *C. carandas*, and the stem and flowers of *C. macrocarpa* (Figure 4).

This review article concludes that crude extracts from different parts of *Carissa* species possess significant anti-inflammatory, antiarthritic, adaptogenic, antidiabetic, antimalarial, antiplasmoidal, anticonvulsant, and antiviral activities in in vitro as well as in vivo conditions. However, the mechanism of action of extracts/phytocompounds for their antioxidant, antimicrobial, anticancer, and cardioprotective potential using different model systems is still required. In most of the in vivo studies, the extracts of *Carissa* species showed significant pharmacological activities (anti-inflammatory, hepatoprotective, antipyretic, antimalarial, and antiviral activities) at a concentration between 100 and 500 mg/kg. Toxicity studies revealed that *C. spinarum* and *C. carandas* could be used at up to a 5000 mg/kg dose level without any harmful effect. The various phytochemicals such as alkaloids, phenolic lignins, terpenoids, tannins, coumarins, saponin, and glycosides in the leaves, seed, root, stem, or the entire plant of *Carissa* species are the reason behind all pharmacological activities. These investigations support the traditional use of the genus *Carissa* to treat several ailments, including inflammation, diabetes, malaria, cold, fever, liver, and heart disease. The richness of the *Carissa* fruits in antioxidants, vitamin C, and minerals validates their use as a food additive in various food preparations. Although several compounds of *Carissa* species (*C. macrocarpa*, *C. bispinosa*, and *C. spinarum*) have been isolated, all these compounds are still not explored for their biological potentials. Thus, the study also concludes that *C. macrocarpa* is the least explored species in terms of extraction of pure compounds. The clinical evaluation of crude extracts and pure compounds from the less explored *Carissa* species in vivo model is still required. Further, the determination of the mechanism of molecular activity of plant extract and its chemical compounds within animal model systems still needs to be explored. The current study is intended to help researchers to acknowledge the therapeutic potential of all plant species of the *Carissa* genus.

## 8. Future Recommendations

The present study recommends exploring the unexplored species of the genus *Carissa,* e.g., *C. boiviniana*, *C. haematocarpa*, *C. pichoniana*, and *C. tetramera,* for their chemical and pharmacological profile. Further analysis of phytoconstituents from *C. bispinosa* is required to obtain new bioactive compounds with significant biological applications. Moreover, clinical evaluation and in vivo models of crude extracts and pure compounds of *Carissa* species are still required to be standardised. Further, the determination of the mechanism of molecular activity of the plant extract and its chemical compounds within animal model systems still needs to be explored.

## Figures and Tables

**Table 1:** Ethnomedicinal uses of *Carissa* species.

*Carissa* Species	Part Used	Indications	References
*C. spinarum*	Leaves	Cold and flu, diabetes, malaria, pneumonia, and childbirth complications	[25]
	Fruits	Constipation, diabetes, heart disease, and obesity	[36]
	Roots	Treat worm-infested wounds in animal	[37]
*C. spinarum* (syn. *C. opaca*)	Leaves	Tanning agent, cure fever and asthma	[22,38]
	Fruits	Aphrodisiac	[38]
	Whole plant	Eye disorder, horn injuries, and maggot wounds in animal	
*C. spinarum* (syn. *C. lanceolata*)	Bark and twig	Toothache, respiratory infections, and cleaning sores	[39]
	Leaves	Mosquito repellent	
*C. spinarum* (syn. *C. edulis*)	Whole plant	Headache, chest complaints, rheumatism, gonorrhoea, syphilis, rabies, and epilepsy	[3,4,5]
		Fever, sickle cell anaemia, and hernia	[5]
	Leaves and roots	Anthelmintic, antiscorbutic, astringent, regulate blood glucose level, stomach pain, and tooth pain	[11]
*C. carandas*	Roots	Stomachic, vermifuge, remedy for itch, and insect repellent	[20,24]
	Stem	Strengthens tendons	[40]
	Leaves	Fevers, earache, diarrhoea, and diabetic ulcers	[20,26]
	Fruits	Anaemia, anti-scorbutic, biliousness	[40]
	Whole plant	Diarrhoea, anorexia, intermittent fever, mouth ulcer, sore throat, syphilitic pain, burning sensation, scabies, and epilepsy	[41,42]
		Anthelmintic, appetizer, antipyretic, stomach disorders, rheumatism, disease of the brain, biliousness, and biliary dysfunction	[43,44]
*C. macrocarpa*	Fruits	Human immunodeficiency virus (HIV) and hepatitis	[8,45]
	Leaves	Diarrhoea in livestock	[8,46]
	Whole plant	Cough and venereal diseases	[46]
*C. bispinosa*	Roots	Toothache	[47]

**Table 2:** Nutritional profile of *Carissa* fruits.

Nutritional Parameters	*C. spinarum*			*C. carandas*	*C. macrocarpa*	
	*C. spinarum* (syn. *C. opaca*)	*C. spinarum*	*C. spinarum* (syn*. C. edulis*)	*C. carandas*	*C. macrocarpa* (syn. *C. grandiflora*)	*C. macrocarpa*
Country	India, Pakistan	India	Uganda	India	India	Tunisia
Moisture (%)	76.60	81.05	84.6	88.70	78.45	78.83
Ash (%)	1.25, 4.78	2.46	-	0.78, 0.0018	0.43	0.5
Total protein (%)	1.30, 6.31	2.07	0.3	2, 2.14	0.56	0.74
Dietary fibre (%)	3.40, 13.55	-	2.65	1.81, 15.64	0.91	-
Crude lipids (%)	0.02	1.30	-	10	1.03	3.53
Carbohydrates (%)	17.39, 47	18.66	-	67, 0.019	-	16.40
Organic matter (%)	98.75	-	-	-	-	-
Total sugar (g)	-	-	-	11.58, 0.04	0.12	-
Non-reducing sugar (mg)	-	-	-	4.25	-	-
Reducing sugar (mg)	-	-	-	41.25; 41.5	-	-
Ascorbic acid (mg/100 g)		-	3.24	62.93	10	100
Sodium (mg/100 g)	-	-	1.79	-	-	-
Calcium (mg/100 g)	1	-	10	1.60, 2.92	-	-
Magnesium (mg/100 g)	8.4	-	4	5.2	-	-
Potassium (mg/100 g)	1.98	-	198	-	-	-
Phosphorus (mg/100 g)	0.24	-	24	24.15	-	-
Iron (mg/100 g)	0.56	-	0.56	3.9, 1.088	-	-
References	[10,22]	[36]	[51]	[49,52,53,54]	[19]	[46]

**Table 3:** Antioxidant potential of *Carissa* species.

*Carissa* Species	IC_50_/EC_50_/SC_50_	Plant Parts	Solvent Used/Compounds	Major Findings	Reference
**DPPH Assay**					
*C. spinarum* (syn. *C. opaca*)	EC_50_ (µg/mL)	Leaves	Methanol, butanol, chloroform, hexane, ethyl acetate, aqueous	38–500,000	[74]
		Fruits		59–250	[75]
		Roots		290–5530	[17]
		Leaves	Methanol	499.95	[76]
*C. spinarum* (syn. *C. edulis*)	IC_50_ (µg/mL)	Fruits	Petroleum ether, ethyl acetate, chloroform, ethanol, water	169–488	[77]
	SC_50_ (µg)	Stem	Chloroform	47.04	[78]
*C. spinarum*	IC_50_ (µg/mL)	Root bark	Chloroform, petroleum ether, dichloromethane, ethyl acetate, *n*-butanol	31.8–500	[74]
*C. carandas*	EC_50_ (µg/mL)	Leaves	Methanol	630.4	[79]
				10.5	[80]
	IC_50_ (µg/mL)	Leaves	Ethanol	1.292	[44]
				1.47	[76]
			Methanol	73.1	[81]
		Fruits		27.4	[52]
		Leaves and fruits	Chloroform and ethanol	195.8–259.5	[82]
*C. macrocarpa*	EC_50_ (µg/mL)	Fruit	Hydroethanolic	9900	[46]
		Leaves		26	[83]
		Stem		281	
*C. bispinosa*	IC_50_	Fruits	Fraction 1 (R_f =_ 0.11)	127.50	[9]
			Fraction 2 (R_f_ = 0.38)	183.38	
**ABTS assay**					
*C. spinarum* (syn. *C. opaca*)	EC_50_ (µg/mL)	Leaves	Methanol, butanol, chloroform, hexane, ethyl acetate, aqueous	70–187	[84]
		Fruits		80–500	[75]
*C. carandas*		Leaves	Methanol	1.75	[80]
**Phsophomolybdate or total antioxidant assay**					
*C. spinarum* (syn. *C. opaca*)	EC_50_ (µg/mL)	Leaves	Methanol, butanol, chloroform, hexane, ethyl acetate, aqueous	30–250	[74]
		Fruits		86–500	[75]
**Thiobarbituric assay**					
*C. spinarum* (syn. *C. edulis*)	IC_50_ (µg/mL)	Fruits	Petroleum ether, ethyl acetate, chloroform, ethanol, water	114–289	[77]
*C. macrocarpa*	EC_50_ (µg/mL)	Fruit	Hydroethanolic	1230	[46]
		Leaves		15.4	[83]
		Stem		12.1	
**β-carotene assay**					
*C. spinarum* (syn. *C. opaca*)	EC_50_ (µg/mL)	Leaves	Methanol, butanol, chloroform, hexane, ethyl acetate, aqueous	145–250	[74]
		Fruits		233.8–8000	[75]
*C. macrocarpa*	EC_50_ (µg/mL)	Fruit	Hydroethanolic	880	[46]
		Leaves		300	[83]
		Stem		270	
**Hydrogen peroxide scavenging activity**					
*C. spinarum* (syn. *C. opaca*)	EC_50_ (µg/mL)	Leaves	Methanol, butanol, chloroform, hexane, ethyl acetate, aqueous	19–250	[74]
		Fruits		47.2–250	[75]
*C. spinarum* (syn. *C. edulis*)	IC_50_ (µg/mL)	Fruits	Petroleum ether, ethyl acetate, chloroform, ethanol, water	138–503	[74]
*C. carandas*	IC_50_ (µg/mL)	Leaves	Ethanol	2.038	[44]
			*n*-Hexane	1.802	
			Methanol	84.0	[81]
**Superoxide radical scavenging activity**					
*C. spinarum* (syn. *C. opaca*)	EC_50_ (µg/mL)	Leaves	Methanol, butanol, chloroform, hexane, ethyl acetate, aqueous	93–206	[74]
		Fruits		33.43–250	[75]
**Hydroxyl radical scavenging activity**					
*C. spinarum* (syn. *C. opaca*)	EC_50_ (µg/mL)	Leaves	Methanol, butanol, chloroform, hexane, ethyl acetate, aqueous	18–22	[74]
		Fruits		36.24–250	[75]
*C. carandas*		Leaves	Methanol (E)	606.9	[79]
**Chelating power**					
*C. spinarum* (syn. *C. opaca*)	EC_50_ (µg/mL)	Leaves	Methanol, butanol, chloroform, hexane, ethyl acetate, aqueous	16–137	[74]
		Fruits		25.1–46.3	[75]
**Reducing power assay**					
*C. spinarum* (syn. *C. edulis*)	IC_50_ (µg)	Fruits	Petroleum ether, ethyl acetate, chloroform, ethanol, water	109–240	[77]
*C. carandas*	EC_50_ (µg/mL)	Fruits	Fruit wine	110300	[85]
		Leaves	Methanol	62.09	[79]
*C. macrocarpa*	EC_50_ (µg/mL)	Fruit	Hydroethanolic	1590	[46]
		Leaves		36	[83]
		Stem		33	
**Isolated compounds of genus *Carissa***					
**DPPH assay**					
*C. spinarum*	IC_50_ _(_µM)	Stem	Olivil	18.1	[23]
			Carinol	20.2	
			Secoisolariciresinol	26.2	
			Carissanol	33.4	
			Cycloolivil	33.2	
			Nortrachelogenin	35.8	
			Pinoresinol	43.4	
			(+)-8-Hydroxypinoresinol	69.5	
	IC_50_ _(_µM)	Root bark	Isolariciresinol3a-*O*-β-d-glucopyranoside	16.5	[73]
			Protocatechuic acid	45.7	
*C. spinarum*	SC_50_ (µM)	Stem	Carissanol	37.12	[78]
			Carinol	47.87	
*C. carandas*	IC_50_ _(_µM)	Stem	Carandoside	116.5	[56]
			(6*S*,7*R*,8*R*)-7a-[(β-glucopyranosyl)oxy]lyoniresinol	21.5	
			(6*R*,7*S*,8*S*)-7a-[(β-glucopyranosyl)oxy]lyoniresinol	43	
			Carissanol	12.7	
			Nortrachelogenin	30.2	
	EC_50_ (µM)	Leaves	Naringin	11.2	[70]
**Superoxide radical scavenging activity**					
*C. carandas*	EC_50_ (µM)	Leaves	Naringin	0.08	[70]

(EC_50_) Half maximal effective concentration. (IC_50_) Inhibitory concentration required for 50% inhibition. (SC_50_) Scavenging concentration required for 50% scavenging.

**Table 4:** Antimicrobial potential of *Carissa* species.

*Carissa* Species	Plant Part Used	Extract/Compound	Microorganisms	MIC (mg/mL)	References
*C. spinarum* (syn. *C. opaca*)	Roots	Ethyl acetate	*Pseudomonas aeruginosa,* *Bacillus subtilis*	0.007–0.008	[35]
*C. spinarum*	Leaves and roots	Methanol and ethanol	*Escherichia coli,* *Staphylococcus aureus*	0.312–2.5	[86]
*C. spinarum* (syn. *C.* *lanceolata*)	Roots	Root bark methanol, root bark dichloromethane, root wood methanol, root wood, dichloromethane	*Bacillus subtilis,* *Escherichia coli*	2.5–20	[39]
*C. carandas*	Fruits	Dichloromethane	*Staphylococcus aureus,* *Escherichia coli,* *Klebsiella pneumonia,* *Enterococcus faecalis*	0.31–5	[87]
	Leaves, stems, and roots	Petroleum ether, water, and methanol	*Bacillus subtilis,* *Agrobacterium tumifaciens,* *Pseudomonas aeruginosa*	0.078–1.25	[88]
*C. macrocarpa*	Fruits	Hydroethanolic	*Escherichia coli,* *Pseudomonas aeruginosa,* *Enterococcus faecalis*	10–20	[46]
	Leaves, stems, and flower	Hydroethanolic	*Escherichia coli,* *Enterococcus faecalis, Listeria monocytogenes*	0.62–20	[84]
	Fruits, stems, and flowers	Essential oil	*Salmonella enterica,* *Staphylococcus aureus,* *Bacillus subtilis*	0.46 -7.5	[71]
*C. macrocarpa* (Syn. *C.* *grandiflora*)	Stems, roots, and leaves	Methanol, *n*-butanol, ethyl acetate, chloroform, *n*-hexane	*Staphylococcus aureus,* *Escherichia coli,* *Staphylococcus* *epidermidis*	0.24–2.69	[89]
**Antifungal activity of *Carissa* species**					
*C. spinarum* (syn. *C. opaca*)	Roots	Ethyl acetate, acetone	*Candida albicans,* *Alternaria solani,* *Aspergillus flavus*	0.05–0.1	[34]
	Roots	Ethyl acetate	*Candida albicans*	0.007	[7]
*C. macrocarpa*	Fruits	Essential oil	*Candida albicans*	0.46	[15]
**Antibacterial activity of some isolated compounds from *Carissa* species**					
*C. spinarum* (syn. *C.* *lanceolata*)	Wood	Dehydrocarissone (15)	*Staphylococcus aureus,* *Escherichia coli,* *Pseudomonas aeruginosa*	0.5–2	[1]
		Carindone (16)		0.5–1	
		Carissone (3)		0.1–2	
*C. spinarum* (syn. *C.* *lanceolata*)	Roots	2-Hydroxyacetophenone (13)	*Bacillus subtilis,* *Staphylococcus aureus,* *Escherichia coli,* *Pseudomonas aeruginosa*	1.25	[39]
		Carinol (6)		1.25	
		Carissone (3)		5–10	
*C. macrocarpa*	Fruits	3β-Hydroxyolean-11-en-28,13 β-olide (14)	*Escherichia coli,* *Enterococcus faecium,* *Staphylococcus* *saprophyticus,* *Klebsiella pneumonia,* *Pseudomonas aeruginosa,* *Staphylococcus aureus*	0.06–0.12	[8]

**Table 5:** In vitro cytotoxic activity of *Carissa* species.

Species	Part Used	Solvent Used	Isolated Compounds	Cell Lines	Major Findings	References
**In vitro** **cytotoxic activity of *Carissa* species (IC_50_ in µg/mL)**						
*C. carandas*	Fruits	Methanol	-	Cervical cell line (Hela), Breast cancer (MCF7), Hepatocellular carcinoma (HeptG2), Bone sarcoma (MG-63)	56.72–86.91	[90]
	Leaves	Methanol		Kidney carcinoma (A-498), Prostate carcinoma (PC-3), Embryonic lung tissue (L-132)	55.56–240	[91]
*C. spinarum*	NM	*n*-Hexane, chloroform, methanol		Melanoma cells (A375)	40–100	[92]
	Stems	NM	Carissanol (**5**)	Normal human (WI-38), Human lung (A549), Human breast (MCF7)	6–17	[23]
			Carinol (**6**)		<1	
			Nortrachelogenin (**23**)		29–100	
*C. spinarum* (syn. *C. congesta*)	Stems	*n*-Butanol		Leukaemia cells (HL-60)	34.58	[93]
*C. spinarum*(Syn. *C. edulis*)	Fresh fruits	Ethanol		Lung cancer (A549)	405	[77]
**In vitro** **cytotoxic activity of *Carissa* species (IC_50_ in µM)**						
*C. spinarum* (syn. *C. edulis*)	Root bark	Methanol	Carissaedulosides A (**24**)	Human leukaemia (HL-60), Lung cancer (A549), Breast cancer (MCF-7), Colon cancer (SW480)	10–20	[94]
			Carissaedulosides D (**25**)		10–22	
			Carissaedulosides J (**26**)		3.8–17	
			Sarhamnoloside (**22**)		0.02–0.13	
			[(1*S*,2*S*,3*S*)-1,2,3,4-tetrahydro-3,7-dihydroxy-1-(4-hydroxy-3 methoxyphenyl)-3 (hydroxymethyl)-6-methoxy-2-naphthalen-yl] methyl β-d-glucopyranoside (**51**)		5.6–19	
*C. carandas*	Leaves	NM	Carandinol (**17**)	HeLa (Cervical cancer), PC-3 (Prostate cancer), 3T3 (Normal mouse fibroblast)	6.87–12.60	[41]
	Fresh leaves	NM	β-sitosterol-3-*O*-β-d-glucopyranoside (**55**)	Small cell lung carcinoma (NCI-H460), Oral squamous cell carcinoma (Cal-27), Normal mouse fibroblast	18.6–63.3	[95]
*C. macrocarpa*	Leaves	Methanol	Kaempferol 3-*O*-robinobioside (**42**)	Human lung cancer (A549)	93.6	[71]
			Kaempferol-3-*O*-α-l-rhamnopyranosyl (1-6)(4′′-*p*-coumaro-yl)β-D-galacto-pyranoside7-*O*-α-l-rhamno-pyranoside (**45**)		100.4	
			Variabiloside E (**46**)		84.3	
**In vitro** **cytotoxic activity of *Carissa* species (Inhibition in %)**						
*C. spinarum* (syn. *C. opaca*)	Leaves	Chloroform, ethyl acetate, methanol	-	Breast cancer (MCF-7)	78–99	[60]
*C. carandas*	Fruits	Aqueous ethanol	-	HeLa cancer cell	67.87	[87]
		Methanol	-	Breast (MCF-7), Colon (HCT-116), Lung (A-549), Ovarian (OVCAR-5), Prostate (PC-3)	63–100	[96]
**In vitro** **cytotoxic activity of *Carissa* species (EC_50_ in µg/mL)**						
*C. carandas*	Leaves	Chloroform	-	Human ovarian carcinoma (Cavo 3)	7.702	[97]
	unripe fruits	*n*-Hexane	-	Lung cancer (NCL)	2.492	
**In vitro** **cytotoxic activity of *Carissa* species (GI_50_ in µg/mL)**						
*C. spinarum* (syn. *C. congesta*)	Roots	Petroleum ether		Breast cancer (MCF7)	18.1	[98]
*C. macrocarpa*	Leaves, stems, and flowers	Hydroethanolic		Breast carcinoma (MCF-7), Cervical carcinoma(HeLa), Non-small cell lungcarcinoma (NCI-H460), Hepatocellular carcinoma (HepG2)	52–400	[83]
	Fruits	-			57–400	[46]

(-) Not reported.

## Abbreviations

EC_50_	Half maximal effective concentration
IC_50_	Inhibitory concentration required for 50% inhibition
SC_50_	Scavenging concentration required for 50% scavenging
GI_50_	Growth inhibition
LD_50_	Lethal dose
MIC	Minimum inhibitory concentration
mg/mL	Milligrams per millilitre
mg/kg	Milligrams per kilogram
µM	Micromolar
µg/mL	Microgram per millilitre
mol/min/g	Mole per min per gram
g/kg	Gram per kilogram
kg/mL	Kilogram per millilitre
et al	et alia
min.	Minimum
syn.	Synonym
*v*/*v*	Volume per volume
